# 24-week home-based walking program in the early adjuvant setting in breast cancer patients who receive aromatase inhibitor endocrine therapy: lessons learned from the SAKK 95/17 WISE prospective, randomized, multicenter trial

**DOI:** 10.1016/j.breast.2026.104789

**Published:** 2026-04-28

**Authors:** Uwe Güth, Nicolette Hoefnagels, Andreas Müller, Sämi Schär, Laurent Rosset, Mahbiz Nobahar Corke, Michael Schwitter, Andreas Jakob, Cathrin Balmelli, Cornelia Leo, Matthias Fehr, David R. Thorn, Salome Riniker, Amina Chouiter-Djebaili, Jana Musilova, Karin Ribi, Friedemann Honecker, the Swiss Cancer Institute

**Affiliations:** a Brust-Zentrum Zürich, Seefeldstrasse 214, 8008, Zurich, Switzerland; b University of Basel, Faculty of Medicine, Klingelbergstrasse 61, 4056, Basel, Switzerland; c Tumor- und BrustZentrum Ostschweiz, Rorschacher Strasse 286, 9016, St. Gallen, Switzerland; d Cantonal Hospital Winterthur, Department of Medical Oncology, Brauerstrasse 15, 8401, Winterthur, Switzerland; e Swiss Cancer Institute, Effingerstrasse 33, 3008, Bern, Switzerland; f Breast Center Fribourg, P.O. Box, 1708, Fribourg, Switzerland; g Hirslanden Clinique des Grangettes, Geneva Oncology and Hematology Center, Chemin des Grangettes 7, Chêne-Bougeries, 1224, Switzerland; h Cantonal Hospital Graubünden, Department of Medical Oncology, Loëstrasse 170, 7000, Chur, Switzerland; i Hirslanden Medical Center, Tumor Centre, Rain 34, 5000, Aarau, Switzerland; j Breast Center Basel, Caba Center for Oncology, Petersgraben 5, 4051, Basel, Switzerland; k Cantonal Hospital Baden, Breast Center, Im Ergel 1, 5404, Baden, Switzerland; l Cantonal Hospital Frauenfeld, Breast Center Thurgau, Pfaffenholzstrasse 4, 8500, Frauenfeld, Switzerland; m Breast Center Basel, Praxis Onkologie, Dr. Thorn, Lange Gasse 78, 4052, Basel, Switzerland; n Réseau Hospitalier Neuchâtelois, Department of Medical Oncology, Rue Chasseral 20, 2300, La Chaux-de- Fonds, Switzerland; o University Medical Center Hamburg-Eppendorf, Department of Hematology, Oncology and Bone Marrow, Transplantation with Section of Pneumology, Martinistrasee 52, 20251, Hamburg, Germany

**Keywords:** Breast cancer, Endocrine therapy with aromatase inhibitors, Physical activity, Activity tracker, Self-efficacy

## Abstract

**Background:**

Aromatase inhibitor (AI) induced arthralgia/myalgia (AIA) is a frequent side-effect of adjuvant breast cancer (BC) endocrine therapy. We investigated the effect of a user-friendly activity tracker to accompany a 24-week walking program, from the start of adjuvant AI-therapy, on patient AIA symptom burden and medication adherence.

**Methods:**

320 early-stage BC patients were included and randomly allocated to the intervention (arm A, *n* = 158) or control arm (arm B, *n* = 162). The intervention in arm A was aimed at briskly walking outdoors for 30 min continuously with 100 steps/minute for 5 days a week, versus an unspecified recommendation of physical activity (PA) in arm B. The patients wore an activity tracker with a customized display.

**Results:**

The intervention compliance in arm A was high, 94% of patients completed the 24-week intervention phase and 68% reached the recommended intervention PA goal. Median daily number of steps was higher in arm A (8542 vs. 7742; p = 0.015). At the end of the intervention phase, activity levels were higher in the intervention group (79.1% vs. 72.8%). These higher values remained unchanged in the follow-up phase at 12 months (79.1% vs. 69.8%) and at 24 months (69.6% vs. 61.1%). Neither the high incidence of AIA during the intervention phase (A:58.2% vs. B:56.2%) nor the reported pain levels differed between the trial arms.

**Conclusion:**

BC patients are highly motivated to pursue new approaches for health promotion in addition to oncological therapies. Activity trackers with training instructional guidance are useful for maintaining training levels achieved over a longer period of time.

## Highlights

• Our activity program allowed two thirds of patients in the intervention arm to achieve the defined goal of physical activity.
• Breast cancer patients are highly motivated to pursue new approaches for health promotion in addition to oncological therapies.
• Activity trackers with training instructional guidance are easy to use and also useful for maintaining the training levels achieved over a longer period of time.
• In this way, promoting physical activity becomes a prime example of patient empowered self-care.

## 1 Introduction

Sport and physical activity is good for your health. This is widely known fact. Regular physical activity (PA) has been proven to help prevent and treat diseases such as obesity, hypertension, heart conditions, stroke, and diabetes. In addition, it also can improve mental health, quality of life and well-being [1]. National and international associations therefore emphasize the importance to raise public awareness for regular exercise and established fitness programs among the population [[1], [2], [3]]. According to the World Health Organization (WHO), adults should undertake 150-300 min of moderate-intensity, or 75–150 min of vigorous-intensity PA, or some equivalent combination of moderate-intensity and vigorous-intensity aerobic PA, per week [4].

While numerous studies showed that PA improves quality of life, increases physiological fitness, reduces side effects of cancer therapy, fatigue, anxiety and depression in breast cancer (BC) patients (general overview in: [[5], [6], [7], [8]]; BC-related literature in: [[9], [10], [11], [12]]), there is only limited evidence of whether it also reduces the risk of BC-related mortality [12].

However, PA could be particularly important for patients diagnosed with hormone receptor-positive BC. During the past 20 years, for the majority of postmenopausal patients in Western countries, endocrine therapy (ET) with an aromatase inhibitor (AI) for at least 5 years has been considered the standard treatment [13,14]. Joint, bone and muscle pain/stiffness are common side effects of this therapy (“AI-associated musculoskeletal symptoms”) [[15], [16], [17], [18]]. In some cases, arthralgia and/or myalgia are so severe and debilitating that patients prematurely stop therapy [19]. Oncologists reassure their patients that these symptoms are in fact not a sign of progressive bone and joint destruction (e.g., in the sense of osteoarthritis or rheumatoid arthritis), but are “merely” a painful hormone deficiency-related tissue irritation that usually subsides after discontinuation of therapy.

The preventive effect of PA on AI-associated musculoskeletal symptoms, however, remains elusive. In addition, activity programs to reduce AI side effects have so far mostly been rather complex [17]. The primary aim of this trial (working title: “WISE-study”; the acronym stands for Walking Intervention for Symptom Elimination under aromatase inhibitor therapy) was to investigate whether a walking intervention, which is easy to implement in the daily routine, commencing with the start of adjuvant AI therapy, can prevent the occurrence of muscle or joint pain/stiffness in BC patients. Furthermore, the trial assessed the effect of PA on symptom burden in general and upon quality of life in BC patients receiving adjuvant AI therapy. During the follow-up phase, the trial assessed whether this intervention leads to a sustained change in lifestyle regarding activity levels, pain thresholds, and better treatment adherence in the intervention group.

## 2 Methods

The “A 24 weeks activity program in patients with early breast cancer receiving aromatase inhibitor therapy” was a prospective multicenter randomized phase III trial (SAKK 95/17) led by the Swiss Cancer Institute (formerly Swiss Group for Clinical Cancer Research, SAKK) conducted at 33 Swiss breast centers.

Adult female BC patients were eligible for the trial if they had undergone primary surgical procedures of pathologically proven AJCC/TNM stage I-III hormone-receptor-positive BC and were planned to start an adjuvant endocrine therapy with an AI alone (for postmenopausal women) or combined with ovarian suppression with an LHRH agonist (for premenopausal women). Further inclusion criteria were WHO performance status 0-2, patient is fluent in German, Italian, or French and is willing to wear a wrist worn activity tracker for 24 weeks. Patients were excluded from enrollment if they had pre-existing severe medical conditions such as heart or lung problems or musculoskeletal conditions precluding participation in the PA program of moderate walking a total of 150 min per week. Further exclusion criteria were active rheumatoid arthritis, regular intake of pain killers (>1 time per week) and mild, moderate, or severe pain (other than post-operative pain) in the last 24 h due to muscle/joint pain on the Brief Pain Inventory-Short Form (BPI-SF; a 9-item tool which assesses the severity of pain and its impact on functioning) single item "worst pain" ≥3 within 7 days prior to registration.

A total of 384 patients were screened for inclusion; of these, 375 patients were eligible to participate and provided written informed consent. All participants were randomly assigned in a 1:1 ratio to participate in one of these two arms.

- Intervention arm (arm A): Home-based walking program consisting of moderate intensity walking outdoors continuously for at least 30 min a day, 5 days a week for 22 weeks, following a 2-week run-in period. The aim was to walk at a speed of at least 100 steps/minute, resulting in at least 3000 steps within 30 min.
- Control arm (arm B): PA as usual. Patients in the control arm were informed that the trial will capture PA following BC. They were informed about the WHO standard recommendation of 150 min of activity per week, but no information/guidance on how to achieve this target was given.

During the 24-week intervention phase, patients in both groups wore an activity tracker on the wrist with customized display (intervention arm: feedback about performed activity; control arm: no feedback). In this study, the Garmin vivofit™ 4 model was used as the fitness activity tracker.

The primary endpoint of the trial was the incidence of muscle or joint pain/stiffness as measured by the BPI-SF single-item worst pain score [20]. Patients were counted as having “muscle or joint pain/stiffness” if.

a) the BPI-SF worst pain score is ≥ 3 at three or more time points, or
b) the BPI-SF worst pain score is ≥ 3 at two or more consecutive time points, or
c) AI was permanently discontinued because of muscle or joint pain/stiffness.

A switch to another non-steroidal AI (anastrozole or letrozole) or to a steroidal AI (exemestane) did not count as permanent discontinuation.

The secondary endpoints included fatigue, hot flashes, quality of life, intensity of muscle or joint pain/stiffness and its impact on everyday functioning, walking activity, general PA, weight, AI treatment adherence, and survival status.

According to the study protocol, there were in total 11 scheduled visits. The screening visit was followed by eight visits at three-week intervals until week 25 during the intervention phase. During these visits, the activity tracker data was downloaded. After completion of the intervention phase, there were two further follow-up visits after 12 and 24 months, respectively.

During all visits, the patients completed four validated patient-reported outcome (PRO) questionnaires.

- the above-mentioned Brief Pain Inventory-Short Form (BPI-SF),
- EORTC QLQC30; developed by the European Organization for Research and Treatment of Cancer; a 30-item questionnaire used to assess the quality of life for cancer patients by measuring physical, psychological, and social functioning, as well as symptom burden [21],
- EORTC QLQ-BR23; a supplementary 23-item questionnaire module to be employed in conjunction with the QLQ-C30 for BC patients; it incorporates five multi-item scales to assess body image, sexual functioning, systemic therapy side effects, breast symptoms, and arm symptoms [21],
- GPAQ (Global Physical Activity Questionnaire); developed by WHO for PA surveillance, the 16-item tool collects information on PA participation in three settings - activity at work, travel to and from locations, recreational activities as well as sedentary behavior [22].

During the intervention phase and the follow-up visits, the AI intake was documented, and the patients also completed the PRO questionnaire MARS-5 (Medication Adherence Report Scale-5), a tool developed for assessing medication adherence [23].

Of the 375 randomized patients, 55 patients (arm A, *n* = 30; arm B, *n* = 25) were excluded from the full analysis set due to insufficient drug intake and/or missing pain assessments.

As shown in Table 1, Table 2, 320 patients were eligible for the full analysis set; 158 patients were randomized in the intervention arm A and 162 in the control arm B.

**Table 1:** Baseline demographic and clinical characteristics.

Characteristic	Arm A *n* = 158	Arm B *n* = 162	All patients *n* = 320
Median age at registration (years)	60	60	60
Median body mass index	25.4	24.6	25.0
Menopausal status: postmenopausal (%)	137 (86.7)	138 (85.2)	275 (85.9)
AJCC/UICC^a^ TNM stage (%)			
Stage I	111 (70.3)	118 (72.8)	229 (71.6)
Stage II	40 (25.3)	35 (21.6)	75 (23.4)
Stage III	7 (4.4)	9 (5.6)	16 (5.0)
Previous therapy (%)			
Surgery			
Breast conserving therapy	124 (78.5)	125 (77.2)	249 (77.8)
Mastectomy	34 (21.5)	37 (22.8)	71 (22.2)
No axillary staging	8 (5.1)	19 (11.7)	27 (8.4)
Sentinel lymph node biopsy	101 (63.9)	102 (62.7)	203 (63.4)
Axillary lymph node dissection	49 (31.0)	41 (25.3)	90 (28.1)
Previous systemic therapy	68 (43.0)	71 (43.8)	139 (43.4)
Radiotherapy (RT)			
RT excluding axillary lymph nodes	89 (56.3)	89 (54.9)	178 (55.6)
RT including axillary lymph nodes	51 (32.3)	53 (32.7)	104 (32.5)
Screening visit: On how many days per week did the patient physically exercise at least 30 min in a way that the breath and pulse raised noticeably? (%)			
Never	11 (7.0)	13 (8.0)	24 (7.5)
On one to two days per week	52 (32.9)	47 (29.0)	99 (30.9)
On three to four days per week	48 (30.4)	48 (29.6)	96 (30.0)
On five to six days per week	29 (18.3)	26 (16.1)	55 (17.2)
Every day	18 (11.4)	28 (17.3)	46 (14.4)

a AJCC: American Joint Committee on Cancer; UICC: Union internationale contre le cancer.

**Table 2:** Patient disposition at data cutoff.

Trial phase at data cutoff or discontinuation	Arm A *n* = 158	Arm B *n* = 162	All patients *n* = 320
Trial phase at data cutoff or discontinuation (%)			
Intervention phase	4 (2.5)	7 (4.3)	11 (3.4)
Follow-up phase	4 (2.5)	1 (0.6)	309 (96.6)
Visit at data cutoff or discontinuation (%)			
Week 4	1 (0.6)	1 (0.6)	2 (0.6)
Week 7-13 (week 4, 7, and 10)	0	0	0
Week 16-19 (week 16 and 19)	0	2 (1.2)	2 (0.6)
Week 22-25 (week 22 and 25)	3 (1.9)	4 (2.5)	7 (2.2)
Follow-up after 12 months	4 (2.5)	1 (0.6)	5 (1.6)
Follow-up phase after 24 months	150 (95.0)	154 (95.1)	304 (95.0)
Status at 24 months: No evidence of disease	158 (100)	162 (100)	320 (100)

The trial was conducted in compliance with the provisions of the current Declaration of Helsinki and the International Council for Harmonization guidelines for Good Clinical Practice as well as all national legal and regulatory requirements. All patients who participated in this study provided written informed consent. The study protocol was developed by the Swiss Cancer Institute and approved by the research ethics board at each participating institution. The Swiss Cancer Institute was responsible for the collection, maintenance, and analysis of the data.

**Statistical analysis:** All endpoints, except falls and serious adverse events, were analyzed based on the full analysis set. In general, the summary statistics presented for quantitative variables were the median, minimum and maximum values. The summary statistics presented for categorical data were the count and percentage of patients in each category. Endpoints expressed as a score or on a continuous scale collected at several time points were analyzed using a nonparametric rank-based model for longitudinal data. For the primary endpoint, the point estimate and two-sided 95% Clopper-Pearson confidence interval were presented for each arm, and the two treatment arms were compared by a logistic regression model including the treatment arm as independent variable and the stratification factors as strata. A secondary analysis of the primary endpoint was performed, pooling the two randomization groups together and including the mean number of daily minutes spent doing moderate or vigorous intensity activity as independent variable in a logistic regression model. All analyses were performed using the software SAS v. 9.4 and R v. 4.5.0.

## 3 Results

**Fig. 1:**
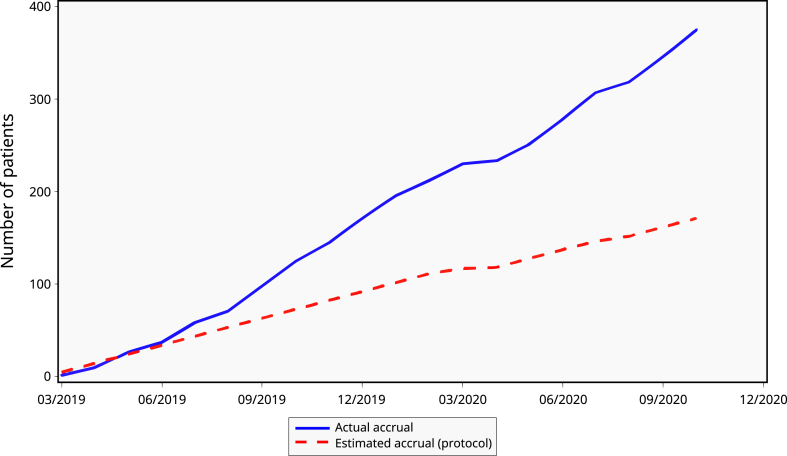
Patient accrual curve. Start of the trial: March 28, 2019; end of accrual: October 10, 2020. The trial ended on December 5, 2022, two years after the randomization of the last patient.

Patient accrual was considerably faster than initially estimated by the protocol (Fig. 1). Within nine months, twice as many patients as planned had been enrolled in the study protocol. This trend continued and by the 18-month mark, the last patient had already been screened.

In study arm A, 68% of patients self-reported to have achieved the set activity goal. During the 24-week intervention phase, median daily number of steps was moderately, yet statistically significantly higher in the intervention arm A (A: 8542 vs. control arm B: 7742; p = 0.015). However, there were no differences between the two study groups in terms of daily duration of activity (median number of minutes; A: 83.1 vs. B: 81.3).

During trial intervention phase, the mean "worst pain" remained continuously between 2 and 3 in both arms during all time points measured. Regarding the trial's primary endpoint, the incidence rates of muscle or joint pain/stiffness were high, but virtually equal (A: 58.3% [95% CI, 50.1%-66.0%] vs. B: 56.2% [95% CI, 48.2%-64.0%], adjusted OR = 1.14 (95% CI, 0.73-1.78), p = 0.577).

In addition, none of the secondary endpoints was significantly different between the trial arms. Adherence to AI over 24 weeks was high in both arms, and only 15 patients (4.7% of the study cohort) discontinued the therapy. Of these, 14 patients (arm A, *n* = 8; arm B, *n* = 6) prematurely stopped therapy due to joint pain/stiffness. One patient reported fatigue as the main reason for non-adherence.

As expected, no serious events were reported during the intervention phase, and no new safety information was captured in either of the two arms.

During trial intervention phase and during follow-up phase at 12 and 24 months, PA was measured by the patient self-report GPAQ, a questionnaire developed for PA surveillance. At the start of the study, the number of participants classified as “active” was even higher in Group B than in Group A (A: 75.3% vs. B: 80.5%). During the intervention phase, activity levels were balanced between the study arms in week 13 (A: 78.5% vs. B: 78.4%), with higher values in the intervention group for the first time at the end (week 25; A: 79.1% vs. B: 72.8%). These higher values for study arm A remained unchanged in the follow-up phase at 12 months (A: 79.1% vs. B: 69.8%) and at 24 months, respectively (A: 69.6% vs. B: 61.1%).

## 4 Discussion

Our trial investigated whether wearing an activity tracker during a 6-months walking program at the start of adjuvant AI therapy had a positive effect on the frequency and intensity of AI-associated muscle or joint pain/stiffness. Although the median number of daily steps in the intervention group increased compared to patients in the control arm, the primary endpoint was not met. The training program failed to reduce symptom burden of musculoskeletal symptoms.

One important reason for not achieving the study objectives is probably that our study cohort recruited Swiss patients exclusively. By international comparison, Swiss women have a lower body mass index (BMI) and a higher level of PA than women in other countries.

- Current data indicates an obesity rate of 9% for Swiss women; this is comparable to that of France and significantly lower than in other European countries, e.g., Sweden (13.7%), the Netherlands (14.5%), Italy: 17.0%, Norway (17.7%), Germany (18.4%), and United Kingdom (27.6%) [24,25].
- In 2022, 73% of Swiss women met national WHO-based PA activity recommendations, a figure that has been increasing over time [[26], [27], [28]]. Women aged 45 and older have shown a particular increase in PA, with many starting well-liked activities such as walking, cycling, swimming, skiing, and jogging, with weight training, yoga, and dance gaining popularity.

We believe that PA programs in countries with higher obesity and inactivity rates, particularly the USA with an obesity rate of 43% [25,[29], [30], [31]], could achieve greater efficiency in terms of reducing AI-associated musculoskeletal symptoms than was the case in our Swiss cohort.

Another explanation for the lack of effectiveness of the activity program upon adherence to AI therapy (one of the secondary endpoints of the study) is the fact that patients were treated in certified BC centers. Previous Swiss data showed that BC patients treated in certified centers had significantly lower non-adherence rates, particularly in the first year of treatment (approximately 5%, as in our trial) [32,33] than is usually reported in the international literature [34]. These low non-adherence rates are probably associated with the fact that most BC patients were treated by specialized doctors experienced with careful management of therapy-related side effects and the ability to carefully advise women through conflicting situations with regards to therapy.

A further reason why the results of the two study groups did not differ significantly is that the overall PA recommendation of 150 min per week and the impact of wearing an activity tracker was also given to the control group and that this awareness might have stimulated PA also in the control group, thus minimizing the effect of the intervention.

The most important lesson we learned from our study was not actually an intentional study objective when planning the trial, namely the strong motivation among our patients towards accepting guidance and support for increased PA in the early phase therapy. While many clinical trials struggle to recruit participants at all, we reached the pre-defined number of participants well before the planned end of recruitment. Patients who were not randomized into the intervention group were disappointed that they were not part of the intervention. The intervention compliance in arm A was high, 94% completed the intervention phase and nearly 70% reached the intervention PA goal per week according to protocol. During the follow-up phase, the patients continued to show a high level of motivation to engage in PA. Once the patients had become accustomed to monitoring their PA with the help of the tracker, their activity levels remained high even two years after the study began.

This shows that patients who are confronted with a cancer diagnosis and treatment are highly motivated to pursue other, new ways of self-care health promotion in addition to completing the recommended oncological therapies [overview in: [35]]. Dealing with challenges presented by the diagnosis of a potentially fatal disease, which usually encompass negative thoughts and feelings, can thus be constructively channeled into positive directions. There are many areas of daily life where changes can be made: in addition to increasing PA, eating a healthier diet, reducing stressful activities and constraints, taking breaks, considering religious and spiritual concerns, participating in community activities, creative and artistic activities, etc. However, the benefits of these self-efficacy activities might not only be directly associated with cancer care but also translate into a reduced risk of comorbidities, improved cardiovascular function and physical fitness and thus improved wellbeing and better daily functioning.

As with many aspects of everyday life, it is important to strike a reasonable balance when making adjustments to current habits. This applies both to patients and to doctors who recommend lifestyle changes. The type and scope of PA should depend on the condition of the individual patient. With women facing a life-threatening illness, in some cases recommending additional PA may seem to be unnecessarily burdensome or too simplistic and can place excessive demands on the patient's time and energy resources [36].

**Conclusion:** Although the primary endpoint (reduction in the frequency and severity of AI-induced arthralgia) was not met, the results of this trial have encouraged us to talk to BC patients about options for further health-promoting measures early on in the adjuvant therapy phase. Which measures are actually taken is then up to the patient. At a time in which older adults are also becoming increasingly familiar with electronic gadgets, we consider activity trackers to be a very suitable means of promoting PA [[37], [38], [39], [40]]. These tools make it easier to get started with structured training in an interactive and playful manner while also maintaining the training levels achieved over a longer period of time. In this way, promoting physical activity becomes a prime example of patient self-efficacy [41,42].

## CRediT authorship contribution statement

**Uwe Güth:** Writing – review & editing, Writing – original draft, Validation, Investigation, Conceptualization. **Nicolette Hoefnagels:** Writing – review & editing, Project administration, Methodology, Investigation, Funding acquisition, Data curation, Conceptualization. **Andreas Müller:** Writing – review & editing, Writing – original draft, Investigation, Conceptualization. **Sämi Schär:** Writing – review & editing, Writing – original draft, Validation, Methodology, Investigation, Formal analysis, Data curation, Conceptualization. **Laurent Rosset:** Writing – review & editing, Writing – original draft, Investigation, Conceptualization. **Mahbiz Nobahar Corke:** Writing – review & editing, Writing – original draft, Investigation, Conceptualization. **Michael Schwitter:** Writing – review & editing, Writing – original draft, Investigation, Conceptualization. **Andreas Jakob:** Writing – review & editing, Writing – original draft, Investigation, Conceptualization. **Cathrin Balmelli:** Writing – review & editing, Writing – original draft, Investigation, Conceptualization. **Cornelia Leo:** Writing – review & editing, Writing – original draft, Investigation, Conceptualization. **Matthias Fehr:** Writing – review & editing, Writing – original draft, Investigation, Conceptualization. **David R. Thorn:** Writing – review & editing, Writing – original draft, Investigation, Conceptualization. **Salome Riniker:** Writing – review & editing, Writing – original draft, Investigation, Conceptualization. **Amina Chouiter-Djebaili:** Writing – review & editing, Writing – original draft, Investigation, Conceptualization. **Jana Musilova:** Writing – review & editing, Writing – original draft, Validation, Project administration, Investigation, Data curation, Conceptualization. **Karin Ribi:** Writing – review & editing, Supervision, Project administration, Methodology, Conceptualization. **Friedemann Honecker:** Writing – review & editing, Writing – original draft, Validation, Supervision, Project administration, Methodology, Investigation, Funding acquisition, Conceptualization.

## Data availability statement

All data generated or analyzed during this study are documented in the “SAKK 95/17: Clinical Study Report for final analysis” prepared by the trial statistician Dr. Sämi Schär. Further information is available upon request to the corresponding author.

## Declaration of generative AI and AI-assisted technologies in the writing process

No AI tools were used in the writing process.

## Funding

The study was funded by 10.13039/100012472Swiss Central Intelligence Agency the following foundations: Ned/Laszlo Foundation, Klara and Erwin Roth-Frei Foundation, 10.13039/501100013362Swiss Cancer Research, 10.13039/501100016009Rising Tide Foundation for Clinical Cancer Research.

## Declaration of competing interest

The authors declare that there are no financial or personal relationships with other people or organizations that could inappropriately influence the work reported or the conclusions, implications, or opinions stated.
