# Supplementary material for: 24-week home-based walking program in the early adjuvant setting in breast cancer patients who receive aromatase inhibitor endocrine therapy: lessons learned from the SAKK 95/17 WISE prospective, randomized, multicenter trial
Source: Breast. 2026 Apr 28;88:104789. doi: 10.1016/j.breast.2026.104789 (PMC13137034; doi:10.1016/j.breast.2026.104789)
Supplement: Multimedia component 1 [file mmc1.docx]

**SAKK 95/17 WISE prospective, randomized, multicenter trial**

**24-week home-based walking program in the early adjuvant setting in breast cancer patients who receive aromatase inhibitor endocrine therapy**

**Detailed Data Analysis Report**

**Efficacy evaluation**

## Efficacy results

### Primary endpoint

#### Primary analysis

The primary endpoint is the incidence of muscle or joint pain/stiffness as measured by BPI-SF single-item worst pain score. Patients were counted as having “muscle or joint pain/stiffness” if:

a) the BPI-SF worst pain score is ≥ 3 at three or more time points, or

b) the BPI-SF worst pain score is ≥ 3 at two or more consecutive time points, or

c) AI was permanently discontinued because of muscle or joint pain/stiffness. Note: Switch to another non-steroidal AI (anastrozole or letrozole) or to a steroidal AI (exemestane) does not count as permanent discontinuation.

Otherwise, patients were counted as not having“muscle or joint pain/stiffness”. Missing values of the BPI-SF worst pain score were treated as the pain score ≥ 3. Details can be found in Table 1 of SAP v1.0.

There were in total 8 scheduled visits (21 days between two visits) for the BPI form during the intervention phase (excluding the one from baseline). In the protocol, only number of visits with worst pain was of focus, the time between visits was not considered. After discussing with the IDMC during the feasibility analysis, we agreed to consider each visit within ± 14 days (including 14 days) of the scheduled visit as a valid visit. Otherwise, the visit would be considered as not performed (missing) so the way of handling missing values mentioned above applies.

A total of 92 (58.2%) patients in Arm A and 91 (56.2%) in Arm B had muscle or joint pain/stiffness. The incidence rate of this event is virtually equal in the intervention arm and the control arm. The Z score is higher than the futility boundary (Table 1). Therefore, the null hypothesis cannot be rejected (P=0.641) and the primary endpoint is negative.

Table 1. Result of primary endpoint

| **Arm A (n=158)** | **Arm B (n=162)** | **Z score** | **Boundary of futility Z score** | **P-value** |
| --- | --- | --- | --- | --- |
| 92 (58.2%) | 91 (56.2%) | 0.361 | -1.64 | 0.641 |

#### Supporting analysis

A supporting analysis of the primary endpoint was performed. The 2 arms were compared using a logistic regression model including the treatment arm as independent variable and the stratification factors as strata. No significant difference between treatment arms was found (Table 2). In addition, a secondary analysis of the primary endpoint was performed pooling the 2 randomization groups together and including the mean number of daily minutes spent doing moderate or vigorous intensity activity as independent variable in a logistic regression model. This analysis was done to investigate the effect of the physical activity on pain/stiffness independently of the randomization group as it can be argued that patients in both groups will increase their daily activity in a trial with this design. There was no significant association between the primary endpoint and the number of daily minutes spent doing moderate or vigorous intensity activity (Table 3). Finally, the mean pain scores for muscle pain/stiffness at its worst over time is shown in Figure 1. Regions of pain during the trial are summarized in Table 4.

Table 2. Result from the stratified logistic model of the primary endpoint

| **Effect** | **Odds ratio (95% CI)** | **P-value** |
| --- | --- | --- |
| Arm A vs Arm B | 1.14 (0.73, 1.78) | 0.577 |

Table 3. Effect of activity on the primary endpoint (logistic regression)

| **Effect** | **Odds ratio (95% CI)** | **P-value** |
| --- | --- | --- |
| No. of daily minutes spent doing moderate or vigorous activity | 0.99 (0.98, 1.01) | 0.274 |

*
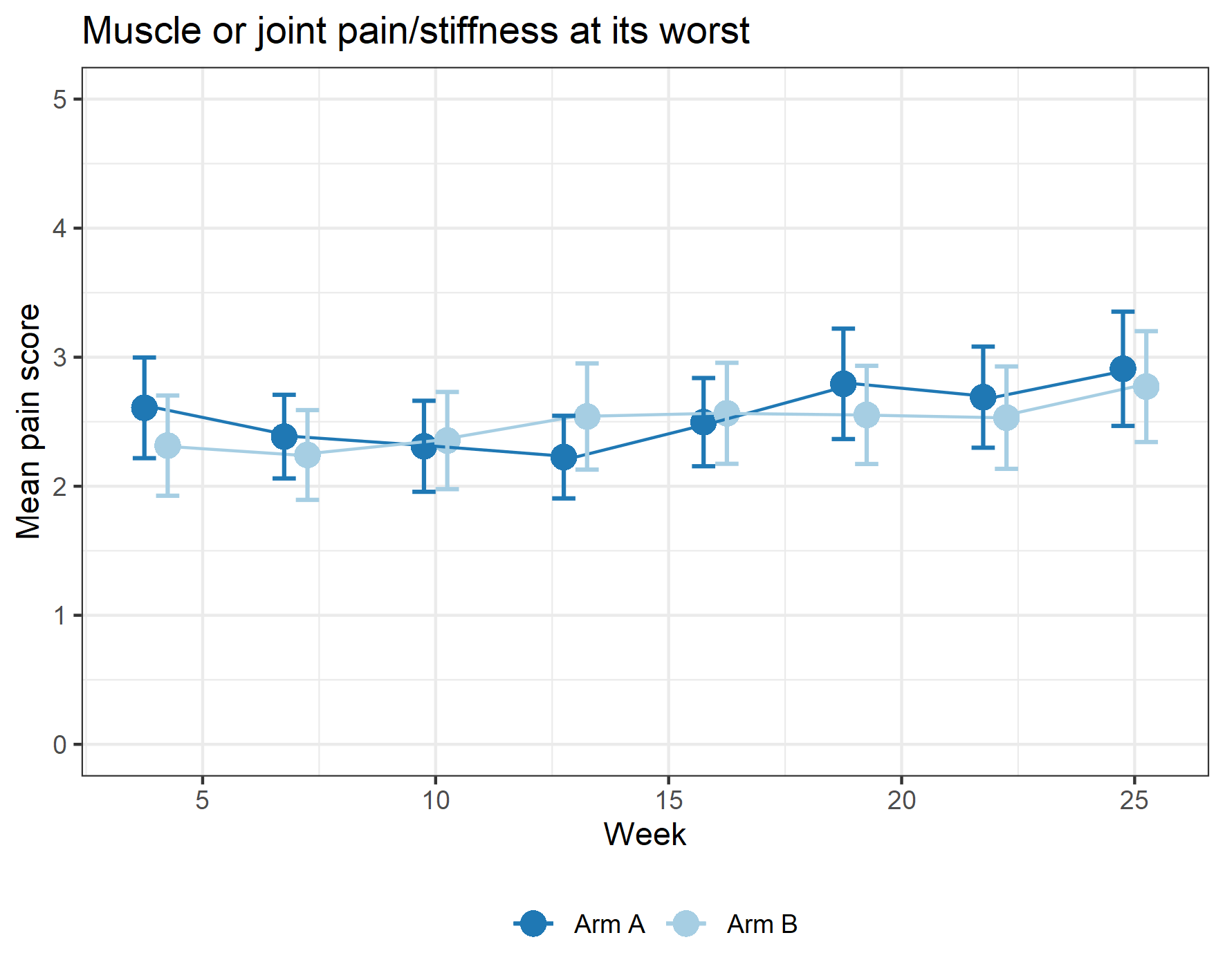
*

Figure 1. Mean pain score for muscle pain/stiffness at its worst over time, by treatment arm

Table 4. Regions of pain

|  | | **Arm A** | | **Arm B** | |
| --- | --- | --- | --- | --- | --- |
| **Region** | **Visit** | **Left (N=158)** | **Right (N=158)** | **Left (N=162)** | **Right (N=162)** |
| Ankle | Eligibility | 68 (43.0%) | 72 (45.6%) | 67 (41.4%) | 69 (42.6%) |
|  | Week 4 | 68 (43.0%) | 72 (45.6%) | 67 (41.6%) | 69 (42.9%) |
|  | Week 7 | 68 (43.6%) | 72 (46.2%) | 67 (41.6%) | 69 (42.9%) |
|  | Week 10 | 68 (43.9%) | 72 (46.5%) | 67 (41.6%) | 69 (42.9%) |
|  | Week 13 | 68 (43.9%) | 72 (46.5%) | 67 (41.6%) | 69 (42.9%) |
|  | Week 16 | 68 (43.9%) | 72 (46.5%) | 67 (41.6%) | 69 (42.9%) |
|  | Week 19 | 68 (43.9%) | 72 (46.5%) | 67 (41.6%) | 69 (42.9%) |
|  | Week 22 | 68 (43.9%) | 72 (46.5%) | 65 (41.1%) | 68 (43.0%) |
|  | Week 25 | 68 (43.6%) | 72 (46.2%) | 64 (40.5%) | 67 (42.4%) |
|  | FU 1 | 66 (42.9%) | 70 (45.5%) | 62 (40.0%) | 65 (41.9%) |
|  | FU 2 | 65 (43.3%) | 70 (46.7%) | 62 (40.3%) | 65 (42.2%) |
| Elbow | Eligibility | 40 (25.3%) | 41 (25.9%) | 46 (28.4%) | 43 (26.5%) |
|  | Week 4 | 40 (25.3%) | 41 (25.9%) | 46 (28.6%) | 43 (26.7%) |
|  | Week 7 | 40 (25.6%) | 41 (26.3%) | 46 (28.6%) | 43 (26.7%) |
|  | Week 10 | 39 (25.2%) | 41 (26.5%) | 46 (28.6%) | 43 (26.7%) |
|  | Week 13 | 39 (25.2%) | 41 (26.5%) | 46 (28.6%) | 43 (26.7%) |
|  | Week 16 | 39 (25.2%) | 41 (26.5%) | 46 (28.6%) | 43 (26.7%) |
|  | Week 19 | 39 (25.2%) | 41 (26.5%) | 46 (28.6%) | 43 (26.7%) |
|  | Week 22 | 39 (25.2%) | 41 (26.5%) | 45 (28.5%) | 42 (26.6%) |
|  | Week 25 | 39 (25.0%) | 41 (26.3%) | 44 (27.8%) | 41 (25.9%) |
|  | FU 1 | 40 (26.0%) | 41 (26.6%) | 42 (27.1%) | 39 (25.2%) |
|  | FU 2 | 39 (26.0%) | 41 (27.3%) | 42 (27.3%) | 38 (24.7%) |
| Foot | Eligibility | 82 (51.9%) | 85 (53.8%) | 70 (43.2%) | 70 (43.2%) |
|  | Week 4 | 82 (51.9%) | 85 (53.8%) | 69 (42.9%) | 70 (43.5%) |
|  | Week 7 | 82 (52.6%) | 85 (54.5%) | 69 (42.9%) | 70 (43.5%) |
|  | Week 10 | 81 (52.3%) | 85 (54.8%) | 69 (42.9%) | 70 (43.5%) |
|  | Week 13 | 81 (52.3%) | 85 (54.8%) | 69 (42.9%) | 70 (43.5%) |
|  | Week 16 | 81 (52.3%) | 85 (54.8%) | 69 (42.9%) | 70 (43.5%) |
|  | Week 19 | 81 (52.3%) | 85 (54.8%) | 69 (42.9%) | 70 (43.5%) |
|  | Week 22 | 81 (52.3%) | 85 (54.8%) | 67 (42.4%) | 69 (43.7%) |
|  | Week 25 | 81 (51.9%) | 85 (54.5%) | 66 (41.8%) | 68 (43.0%) |
|  | FU 1 | 80 (51.9%) | 83 (53.9%) | 65 (41.9%) | 67 (43.2%) |
|  | FU 2 | 79 (52.7%) | 83 (55.3%) | 65 (42.2%) | 67 (43.5%) |
| Hand | Eligibility | 93 (58.9%) | 95 (60.1%) | 96 (59.3%) | 105 (64.8%) |
|  | Week 4 | 93 (58.9%) | 95 (60.1%) | 96 (59.6%) | 104 (64.6%) |
|  | Week 7 | 93 (59.6%) | 95 (60.9%) | 96 (59.6%) | 104 (64.6%) |
|  | Week 10 | 93 (60.0%) | 95 (61.3%) | 96 (59.6%) | 104 (64.6%) |
|  | Week 13 | 93 (60.0%) | 95 (61.3%) | 96 (59.6%) | 104 (64.6%) |
|  | Week 16 | 93 (60.0%) | 95 (61.3%) | 96 (59.6%) | 104 (64.6%) |
|  | Week 19 | 93 (60.0%) | 95 (61.3%) | 96 (59.6%) | 104 (64.6%) |
|  | Week 22 | 93 (60.0%) | 95 (61.3%) | 93 (58.9%) | 101 (63.9%) |
|  | Week 25 | 93 (59.6%) | 95 (60.9%) | 93 (58.9%) | 101 (63.9%) |
|  | FU 1 | 92 (59.7%) | 94 (61.0%) | 90 (58.1%) | 98 (63.2%) |
|  | FU 2 | 90 (60.0%) | 92 (61.3%) | 89 (57.8%) | 97 (63.0%) |
| Hip | Eligibility | 61 (38.6%) | 70 (44.3%) | 57 (35.2%) | 60 (37.0%) |
|  | Week 4 | 61 (38.6%) | 70 (44.3%) | 57 (35.4%) | 60 (37.3%) |
|  | Week 7 | 60 (38.5%) | 69 (44.2%) | 57 (35.4%) | 60 (37.3%) |
|  | Week 10 | 60 (38.7%) | 68 (43.9%) | 57 (35.4%) | 60 (37.3%) |
|  | Week 13 | 60 (38.7%) | 68 (43.9%) | 57 (35.4%) | 60 (37.3%) |
|  | Week 16 | 60 (38.7%) | 68 (43.9%) | 57 (35.4%) | 60 (37.3%) |
|  | Week 19 | 60 (38.7%) | 68 (43.9%) | 57 (35.4%) | 60 (37.3%) |
|  | Week 22 | 60 (38.7%) | 68 (43.9%) | 55 (34.8%) | 58 (36.7%) |
|  | Week 25 | 60 (38.5%) | 68 (43.6%) | 54 (34.2%) | 57 (36.1%) |
|  | FU 1 | 60 (39.0%) | 69 (44.8%) | 52 (33.5%) | 56 (36.1%) |
|  | FU 2 | 58 (38.7%) | 66 (44.0%) | 52 (33.8%) | 56 (36.4%) |
| Knee | Eligibility | 95 (60.1%) | 97 (61.4%) | 95 (58.6%) | 100 (61.7%) |
|  | Week 4 | 95 (60.1%) | 97 (61.4%) | 95 (59.0%) | 100 (62.1%) |
|  | Week 7 | 94 (60.3%) | 96 (61.5%) | 95 (59.0%) | 100 (62.1%) |
|  | Week 10 | 94 (60.6%) | 96 (61.9%) | 95 (59.0%) | 100 (62.1%) |
|  | Week 13 | 94 (60.6%) | 96 (61.9%) | 95 (59.0%) | 100 (62.1%) |
|  | Week 16 | 94 (60.6%) | 96 (61.9%) | 95 (59.0%) | 100 (62.1%) |
|  | Week 19 | 94 (60.6%) | 96 (61.9%) | 95 (59.0%) | 100 (62.1%) |
|  | Week 22 | 94 (60.6%) | 96 (61.9%) | 93 (58.9%) | 98 (62.0%) |
|  | Week 25 | 94 (60.3%) | 96 (61.5%) | 92 (58.2%) | 97 (61.4%) |
|  | FU 1 | 94 (61.0%) | 96 (62.3%) | 89 (57.4%) | 94 (60.6%) |
|  | FU 2 | 93 (62.0%) | 95 (63.3%) | 88 (57.1%) | 93 (60.4%) |
| Shoulder | Eligibility | 73 (46.2%) | 72 (45.6%) | 78 (48.1%) | 73 (45.1%) |
|  | Week 4 | 73 (46.2%) | 72 (45.6%) | 78 (48.4%) | 73 (45.3%) |
|  | Week 7 | 73 (46.8%) | 72 (46.2%) | 78 (48.4%) | 73 (45.3%) |
|  | Week 10 | 73 (47.1%) | 72 (46.5%) | 78 (48.4%) | 73 (45.3%) |
|  | Week 13 | 73 (47.1%) | 72 (46.5%) | 78 (48.4%) | 73 (45.3%) |
|  | Week 16 | 73 (47.1%) | 72 (46.5%) | 78 (48.4%) | 73 (45.3%) |
|  | Week 19 | 73 (47.1%) | 72 (46.5%) | 78 (48.4%) | 73 (45.3%) |
|  | Week 22 | 73 (47.1%) | 72 (46.5%) | 76 (48.1%) | 71 (44.9%) |
|  | Week 25 | 73 (46.8%) | 72 (46.2%) | 76 (48.1%) | 71 (44.9%) |
|  | FU 1 | 72 (46.8%) | 71 (46.1%) | 76 (49.0%) | 70 (45.2%) |
|  | FU 2 | 71 (47.3%) | 70 (46.7%) | 75 (48.7%) | 69 (44.8%) |
| Wrist | Eligibility | 54 (34.2%) | 56 (35.4%) | 56 (34.6%) | 60 (37.0%) |
|  | Week 4 | 54 (34.2%) | 56 (35.4%) | 56 (34.8%) | 60 (37.3%) |
|  | Week 7 | 54 (34.6%) | 56 (35.9%) | 56 (34.8%) | 60 (37.3%) |
|  | Week 10 | 54 (34.8%) | 56 (36.1%) | 56 (34.8%) | 60 (37.3%) |
|  | Week 13 | 54 (34.8%) | 56 (36.1%) | 56 (34.8%) | 60 (37.3%) |
|  | Week 16 | 54 (34.8%) | 56 (36.1%) | 56 (34.8%) | 60 (37.3%) |
|  | Week 19 | 54 (34.8%) | 56 (36.1%) | 56 (34.8%) | 60 (37.3%) |
|  | Week 22 | 54 (34.8%) | 56 (36.1%) | 54 (34.2%) | 58 (36.7%) |
|  | Week 25 | 54 (34.6%) | 56 (35.9%) | 53 (33.5%) | 57 (36.1%) |
|  | FU 1 | 54 (35.1%) | 56 (36.4%) | 51 (32.9%) | 55 (35.5%) |
|  | FU 2 | 54 (36.0%) | 56 (37.3%) | 51 (33.1%) | 55 (35.7%) |

### Secondary endpoints

#### Fatigue

Fatigue was assessed at baseline, during intervention phase at 12 and 24 weeks (± 7 days) and during follow-up phase at 12 and 24 months (± 61 days) by the FA scale of the EORTC QLQ-C30 version 3.0. Three items, Q10, Q12 and Q18, contribute to the FA scale, each item being rated on 1-4 scale [8]. The scale score ranges from 0 to 100. A high score represents a high level of symptomatology/problems. Median and range of the FA scores by treatment arm and visit are shown in Table 5 mean and standard deviation (SD) in Table 6 and mean change from baseline in Table 7 and Figure 2. The FA score was analyzed using analyzed using a nonparametric rank-based model for longitudinal data [3].

There was no significant difference between treatment arms, but the FA score differed significantly between time points (Table 8, Figure 2).

Table 5. Median and range of FA scores by treatment arm and visit

|  | | | **Arm A** | | **Arm B** | |
| --- | --- | --- | --- | --- | --- | --- |
| **Endpoint** | **Score** | **Visit** | **N** | **Median (range)** | **N** | **Median (range)** |
| Fatigue | FA score | Baseline | 155 | 22 (0.0, 100.0) | 162 | 22 (0.0, 100.0) |
|  |  | Week 13 | 151 | 22 (0.0, 88.9) | 157 | 22 (0.0, 100.0) |
|  |  | Week 25 | 150 | 22 (0.0, 100.0) | 152 | 22 (0.0, 100.0) |
|  |  | FU 1 (12 months) | 141 | 22 (0.0, 77.8) | 143 | 22 (0.0, 100.0) |
|  |  | FU 2 (24 months) | 131 | 22 (0.0, 88.9) | 135 | 22 (0.0, 100.0) |

Table 6. Mean and standard deviation (SD) of FA scores by treatment arm and visit

|  | | | **Arm A** | | **Arm B** | |
| --- | --- | --- | --- | --- | --- | --- |
| **Endpoint** | **Score** | **Visit** | **N** | **Mean (SD)** | **N** | **Mean (SD)** |
| Fatigue | FA score | Baseline | 155 | 26.9 (20.5) | 162 | 30.0 (25.3) |
|  |  | Week 13 | 151 | 24.2 (20.0) | 157 | 27.2 (24.6) |
|  |  | Week 25 | 150 | 25.9 (23.5) | 152 | 25.5 (24.7) |
|  |  | FU 1 (12 months) | 141 | 24.7 (19.7) | 143 | 27.2 (24.0) |
|  |  | FU 2 (24 months) | 131 | 24.5 (20.0) | 135 | 26.8 (24.0) |

Table 7. Mean and 95% CI of change of FA scores from baseline by treatment arm and visit

|  | | | **Arm A** | | **Arm B** | |
| --- | --- | --- | --- | --- | --- | --- |
| **Endpoint** | **Score** | **Visit** | **N** | **Mean change from baseline (95% CI)** | **N** | **Mean change from baseline (95% CI)** |
| Fatigue | Change From Baseline | Baseline | 155 | 26.9 (23.6, 30.1) | 162 | 30.0 (26.1, 34.0) |
|  |  | Week 13 | 151 | -2.3 (-5.8, 1.2) | 157 | -3.0 (-7.3, 1.2) |
|  |  | Week 25 | 150 | -0.7 (-5.1, 3.6) | 152 | -4.1 (-8.3, 0.1) |
|  |  | FU 1 (12 months) | 141 | -2.1 (-6.3, 2.0) | 143 | -2.8 (-7.0, 1.4) |
|  |  | FU 2 (24 months) | 131 | -1.7 (-6.2, 2.9) | 135 | -2.8 (-7.4, 1.8) |

Table 8. Results from a nonparametric rank based model for the endpoint fatigue

| **Endpoint** | **Score** | **Effect** | **ANOVA-Type Statistic** | **P-value** |
| --- | --- | --- | --- | --- |
| Fatigue | FA Score | Arm | 0.090 | 0.764 |
|  |  | Time | 3.066 | 0.032 |
|  |  | Arm:Time | 0.465 | 0.686 |


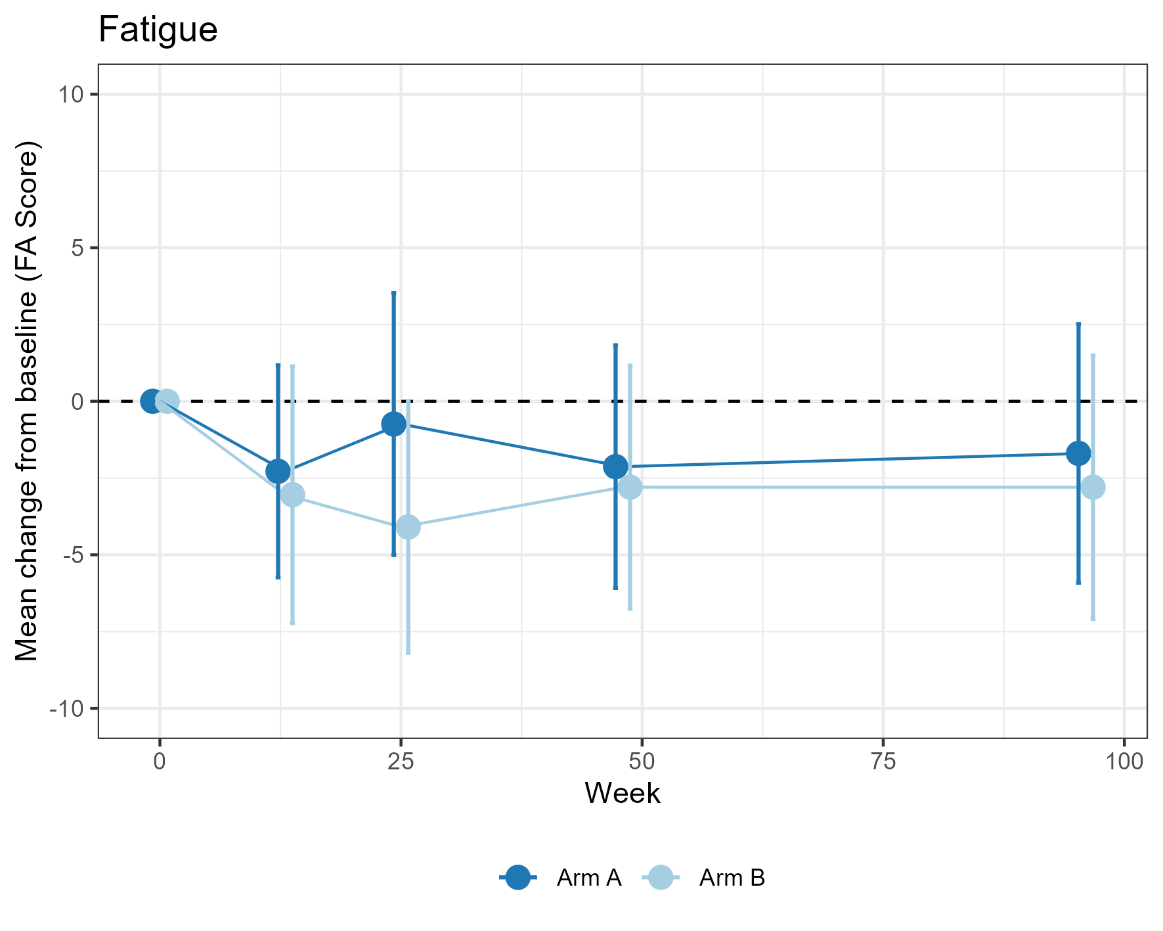


Figure 2. Mean change from baseline and associated 95% CI for FA score by arm and week.

#### Hot flashes

Hot flashes were assessed at baseline, during intervention phase at 12 and 24 weeks (± 7 days), and during follow-up phase at 12 and 24 months (± 61 days) via the transformed item 37 of the EORTC QLQ BR-23. A high score will represent a high level of symptomatology/problems. Median and range of the EORTC QLQ BR-23 by treatment arm and visit are shown in Table 9, mean and SD in Table 10 and mean change and 95% CI from baseline in Table 11 and Figure 3. The QLQ BR-23 score was analyzed using a nonparametric rank-based model for longitudinal data [3].

There was no significant difference between treatment arms, but the QLQ BR-23 score significantly differed between time points Table 12 (Figure 3). Furthermore, there was a significant interaction between arm and time with hot flashes increasing more over time in arm A. This effect may be caused mostly by a large difference between arms at baseline (Table 11).

Table 9. Median and range of EORTC QLQ BR-23 scores by treatment arm and visit

|  | | | **Arm A** | | **Arm B** | |
| --- | --- | --- | --- | --- | --- | --- |
| **Endpoint** | **Score** | **Visit** | **N** | **Median (range)** | **N** | **Median (range)** |
| Hot flashes | EORTC QLQ BR-23 | Baseline | 156 | 0 (0.0, 100.0) | 161 | 33 (0.0, 100.0) |
|  |  | Week 13 | 151 | 33 (0.0, 100.0) | 155 | 33 (0.0, 100.0) |
|  |  | Week 25 | 150 | 33 (0.0, 100.0) | 152 | 33 (0.0, 100.0) |
|  |  | FU 1 (12 months) | 142 | 33 (0.0, 100.0) | 146 | 33 (0.0, 100.0) |
|  |  | FU 2 (24 months) | 133 | 33 (0.0, 100.0) | 136 | 33 (0.0, 100.0) |

Table 10. Mean and SD of EORTC QLQ BR-23 scores by treatment arm and visit

|  | | | **Arm A** | | **Arm B** | |
| --- | --- | --- | --- | --- | --- | --- |
| **Endpoint** | **Score** | **Visit** | **N** | **Mean (SD)** | **N** | **Mean (SD)** |
| Hot flashes | EORTC QLQ BR-23 | Baseline | 156 | 24.1 (30.0) | 161 | 35.4 (33.3) |
|  |  | Week 13 | 151 | 38.0 (31.5) | 155 | 42.8 (33.5) |
|  |  | Week 25 | 150 | 42.2 (32.2) | 152 | 42.3 (33.7) |
|  |  | FU 1 (12 months) | 142 | 39.2 (31.6) | 146 | 41.8 (30.3) |
|  |  | FU 2 (24 months) | 133 | 36.8 (31.9) | 136 | 41.2 (31.7) |

Table 11. Mean and 95% CI of EORTC QLQ BR-23 score change from baseline by treatment arm and visit

|  | | | **Arm A** | | **Arm B** | |
| --- | --- | --- | --- | --- | --- | --- |
| **Endpoint** | **Score** | **Visit** | **N** | **Mean change from baseline (95% CI)** | **N** | **Mean change from baseline (95% CI)** |
| Hot flashes | Change From Baseline | Baseline | 156 | 24.1 (19.4, 28.9) | 161 | 35.4 (30.2, 40.6) |
|  |  | Week 13 | 151 | 14.3 (8.9, 19.8) | 155 | 7.7 (2.6, 12.9) |
|  |  | Week 25 | 150 | 18.2 (12.9, 23.6) | 152 | 7.9 (1.9, 13.8) |
|  |  | FU 1 (12 months) | 142 | 15.7 (10.3, 21.2) | 146 | 8.0 (2.5, 13.4) |
|  |  | FU 2 (24 months) | 133 | 14.0 (8.1, 20.0) | 136 | 6.4 (0.4, 12.3) |

Table 12. Results from a nonparametric rank based model for the endpoint hot flashes

| **Endpoint** | **Score** | **Effect** | **ANOVA-Type Statistic** | **P-value** |
| --- | --- | --- | --- | --- |
| Hot flashes | QLQ BR-23 | Arm | 2.616 | 0.106 |
|  |  | Time | 20.790 | <0.001 |
|  |  | Arm:Time | 3.486 | 0.016 |


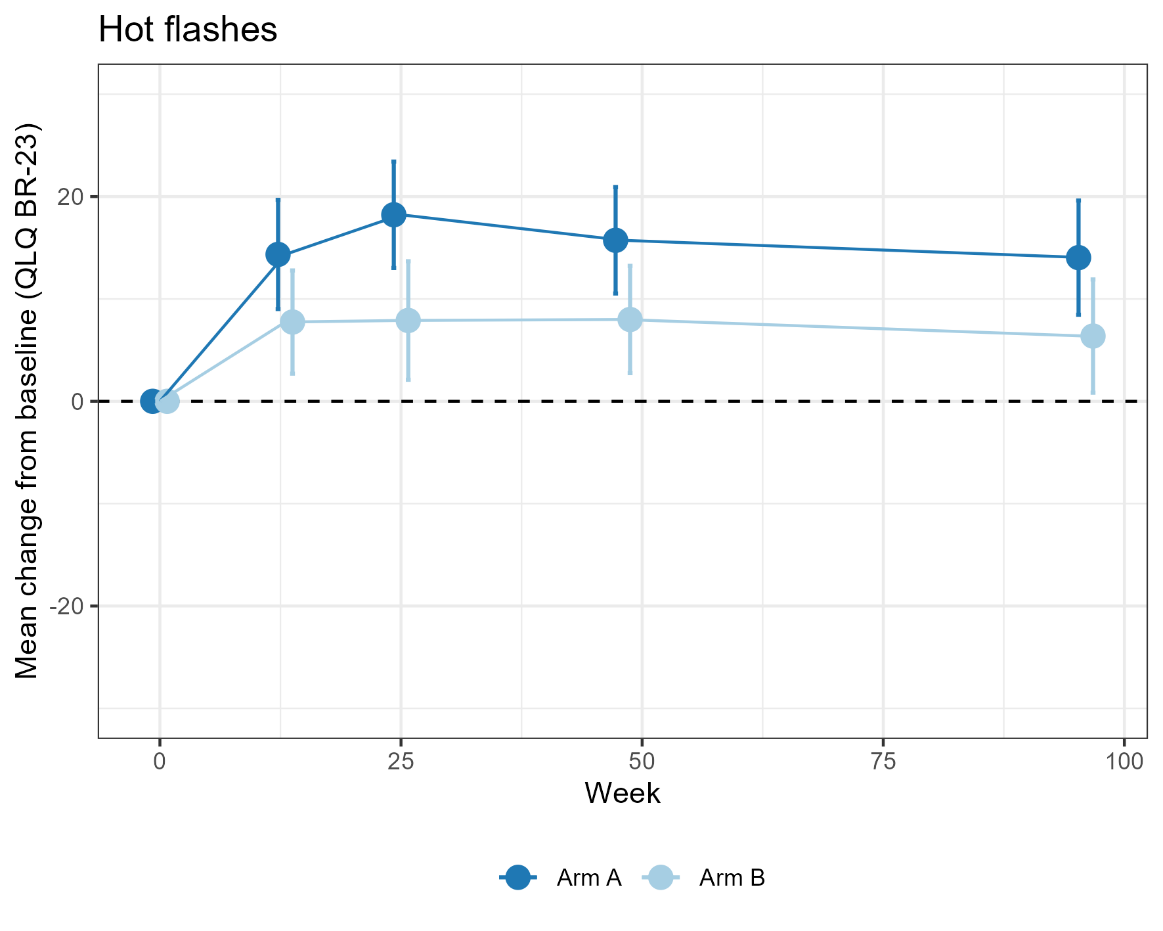


Figure 3. Mean change from baseline and associated 95% CI for the QLQ BR-23 score by arm and week.

#### Quality of Life

QoL was assessed at baseline, during intervention phase at 12 and 24 weeks (± 7 days), and during the follow-up phase at 12 and 24 months (± 61 days) by the EORTC QLQ-C30 version 3.0 complemented with a disease specific module, the QLQ-BR23, measuring additional aspects relevant to patients with breast cancer. The QLQ-C30 is composed of both multi-item scales and single-item measures. These include five functional scales (physical, role, emotional, cognitive and social functioning), three symptom scales (fatigue, nausea/vomiting and pain), a global health status / QoL scale, and six single items (dyspnoea, insomnia, appetite loss, constipation, diarrhoea and financial difficulties). Each of the multi-item scales includes a different set of items - no item occurs in more than one scale. All of the scales and single-item measures range in score from 0 to 100 (after using a linear transformation to standardize the raw score). A high score for a functional scale

represents a high / healthy level of functioning; a high score for the global health status / QoL represents a high QoL, but a high score for a symptom scale / single-item represents a high level of symptomatology / problems. The breast cancer module incorporates five multi-item scales to assess systemic therapy side effects, arm symptoms, breast symptoms, body image and sexual functioning. In addition, single items assess sexual enjoyment, hair loss and future perspective. Scores for all scales were computed according to the EORTC QLQC30 Scoring Manual [10]. Median and range of the EORTC QLQ-C30 items by treatment arm and visit are shown in

Table 13, mean and SD in Table 14 and mean change from baseline and associated 95% Cis in Table 15 and Figure 4, Figure 5 and Figure 6. The EORTC QLQ-C30 scores were analyzed using a nonparametric rank-based model for longitudinal data [3].

There was no significant difference between treatment arms for any of the scores, but the FA score differed significantly between time points for some, namely Global Health Status, Physical functioning, Role Functioning, Emotional Functioning, Fatigue, Pain, Dyspnoea, Insomnia, Diarrhoea, Body image, Future perspective, Breast symptoms and upset by hair loss (Table 16).

Table 13. Median and range of the EORTC QLQ-C30 items by treatment arm and visit

|  | | | **Arm A** | | **Arm B** | |
| --- | --- | --- | --- | --- | --- | --- |
| **Item** | **Score** | **Visit** | **N** | **Median (range)** | **N** | **Median (range)** |
| **Global health status / QoL** | | | | | | |
| Global health status | QL2 | Baseline | 156 | 75 (25.0, 100.0) | 161 | 75 (0.0, 100.0) |
|  |  | Week 13 | 151 | 83 (16.7, 100.0) | 158 | 75 (0.0, 100.0) |
|  |  | Week 25 | 151 | 83 (0.0, 100.0) | 155 | 83 (8.3, 100.0) |
|  |  | FU 1 (12 months) | 142 | 83 (16.7, 100.0) | 147 | 83 (16.7, 100.0) |
|  |  | FU 2 (24 months) | 134 | 75 (16.7, 100.0) | 137 | 83 (16.7, 100.0) |
| **Functional scales** | | | | | | |
| Physical functioning | PF2 | Baseline | 156 | 93 (60.0, 100.0) | 161 | 93 (26.7, 100.0) |
|  |  | Week 13 | 149 | 93 (40.0, 100.0) | 156 | 93 (13.3, 100.0) |
|  |  | Week 25 | 146 | 93 (26.7, 100.0) | 151 | 93 (26.7, 100.0) |
|  |  | FU 1 (12 months) | 142 | 93 (26.7, 100.0) | 146 | 93 (6.7, 100.0) |
|  |  | FU 2 (24 months) | 129 | 93 (26.7, 100.0) | 133 | 93 (26.7, 100.0) |
| Role functioning | RF2 | Baseline | 158 | 100 (0.0, 100.0) | 161 | 83 (0.0, 100.0) |
|  |  | Week 13 | 150 | 100 (16.7, 100.0) | 158 | 100 (0.0, 100.0) |
|  |  | Week 25 | 151 | 100 (0.0, 100.0) | 154 | 100 (0.0, 100.0) |
|  |  | FU 1 (12 months) | 142 | 83 (0.0, 100.0) | 147 | 100 (0.0, 100.0) |
|  |  | FU 2 (24 months) | 132 | 83 (0.0, 100.0) | 136 | 100 (16.7, 100.0) |
| Emotional functioning | EF | Baseline | 155 | 83 (16.7, 100.0) | 158 | 79 (8.3, 100.0) |
|  |  | Week 13 | 150 | 83 (0.0, 100.0) | 157 | 83 (0.0, 100.0) |
|  |  | Week 25 | 149 | 83 (25.0, 100.0) | 153 | 92 (8.3, 100.0) |
|  |  | FU 1 (12 months) | 142 | 83 (8.3, 100.0) | 144 | 83 (8.3, 100.0) |
|  |  | FU 2 (24 months) | 131 | 83 (16.7, 100.0) | 133 | 83 (16.7, 100.0) |
| Cognitive functioning | CF | Baseline | 153 | 100 (33.3, 100.0) | 161 | 100 (0.0, 100.0) |
|  |  | Week 13 | 151 | 100 (33.3, 100.0) | 157 | 100 (0.0, 100.0) |
|  |  | Week 25 | 149 | 83 (16.7, 100.0) | 155 | 100 (16.7, 100.0) |
|  |  | FU 1 (12 months) | 141 | 100 (16.7, 100.0) | 143 | 100 (0.0, 100.0) |
|  |  | FU 2 (24 months) | 133 | 83 (33.3, 100.0) | 135 | 100 (0.0, 100.0) |
| Social functioning | SF | Baseline | 155 | 100 (0.0, 100.0) | 162 | 83 (16.7, 100.0) |
|  |  | Week 13 | 150 | 100 (16.7, 100.0) | 157 | 100 (0.0, 100.0) |
|  |  | Week 25 | 151 | 100 (0.0, 100.0) | 154 | 100 (16.7, 100.0) |
|  |  | FU 1 (12 months) | 142 | 100 (33.3, 100.0) | 144 | 100 (16.7, 100.0) |
|  |  | FU 2 (24 months) | 131 | 100 (0.0, 100.0) | 137 | 100 (33.3, 100.0) |
| Body image | BRBI | Baseline | 155 | 92 (0.0, 100.0) | 160 | 92 (0.0, 100.0) |
|  |  | Week 13 | 150 | 92 (0.0, 100.0) | 155 | 100 (0.0, 100.0) |
|  |  | Week 25 | 149 | 92 (0.0, 100.0) | 153 | 100 (0.0, 100.0) |
|  |  | FU 1 (12 months) | 140 | 92 (8.3, 100.0) | 144 | 100 (0.0, 100.0) |
|  |  | FU 2 (24 months) | 131 | 92 (8.3, 100.0) | 135 | 100 (16.7, 100.0) |
| Emotional functioning | BRSEF | Baseline | 153 | 83 (0.0, 100.0) | 154 | 83 (0.0, 100.0) |
|  |  | Week 13 | 146 | 67 (0.0, 100.0) | 146 | 83 (0.0, 100.0) |
|  |  | Week 25 | 141 | 67 (0.0, 100.0) | 141 | 67 (0.0, 100.0) |
|  |  | FU 1 (12 months) | 133 | 67 (0.0, 100.0) | 133 | 83 (0.0, 100.0) |
|  |  | FU 2 (24 months) | 122 | 67 (0.0, 100.0) | 121 | 83 (0.0, 100.0) |
| Sexual enjoyment | BRSEE | Baseline | 69 | 33 (0.0, 100.0) | 64 | 33 (0.0, 100.0) |
|  |  | Week 13 | 82 | 33 (0.0, 100.0) | 76 | 67 (0.0, 100.0) |
|  |  | Week 25 | 80 | 33 (0.0, 100.0) | 69 | 33 (0.0, 100.0) |
|  |  | FU 1 (12 months) | 78 | 33 (0.0, 100.0) | 63 | 33 (0.0, 100.0) |
|  |  | FU 2 (24 months) | 70 | 33 (0.0, 100.0) | 54 | 33 (0.0, 100.0) |
| Future perspective | BRFU | Baseline | 156 | 67 (0.0, 100.0) | 159 | 67 (0.0, 100.0) |
|  |  | Week 13 | 150 | 67 (0.0, 100.0) | 158 | 67 (0.0, 100.0) |
|  |  | Week 25 | 151 | 67 (0.0, 100.0) | 153 | 67 (0.0, 100.0) |
|  |  | FU 1 (12 months) | 142 | 67 (0.0, 100.0) | 144 | 67 (0.0, 100.0) |
|  |  | FU 2 (24 months) | 134 | 67 (0.0, 100.0) | 136 | 67 (0.0, 100.0) |
| **Symptom scales / items** | | | | | | |
| Fatigue | FA | Baseline | 155 | 22 (0.0, 100.0) | 162 | 22 (0.0, 100.0) |
|  |  | Week 13 | 151 | 22 (0.0, 88.9) | 157 | 22 (0.0, 100.0) |
|  |  | Week 25 | 150 | 22 (0.0, 100.0) | 152 | 22 (0.0, 100.0) |
|  |  | FU 1 (12 months) | 141 | 22 (0.0, 77.8) | 143 | 22 (0.0, 100.0) |
|  |  | FU 2 (24 months) | 131 | 22 (0.0, 88.9) | 135 | 22 (0.0, 100.0) |
| Nausea and vomiting | NV | Baseline | 157 | 0 (0.0, 33.3) | 162 | 0 (0.0, 50.0) |
|  |  | Week 13 | 151 | 0 (0.0, 100.0) | 158 | 0 (0.0, 50.0) |
|  |  | Week 25 | 152 | 0 (0.0, 33.3) | 154 | 0 (0.0, 50.0) |
|  |  | FU 1 (12 months) | 141 | 0 (0.0, 50.0) | 145 | 0 (0.0, 66.7) |
|  |  | FU 2 (24 months) | 132 | 0 (0.0, 50.0) | 136 | 0 (0.0, 50.0) |
| Pain | PA | Baseline | 157 | 17 (0.0, 100.0) | 160 | 17 (0.0, 100.0) |
|  |  | Week 13 | 150 | 17 (0.0, 100.0) | 156 | 17 (0.0, 100.0) |
|  |  | Week 25 | 152 | 17 (0.0, 100.0) | 152 | 17 (0.0, 100.0) |
|  |  | FU 1 (12 months) | 140 | 33 (0.0, 100.0) | 144 | 17 (0.0, 100.0) |
|  |  | FU 2 (24 months) | 131 | 33 (0.0, 100.0) | 135 | 17 (0.0, 100.0) |
| Dyspnoea | DY | Baseline | 157 | 0 (0.0, 66.7) | 162 | 0 (0.0, 100.0) |
|  |  | Week 13 | 151 | 0 (0.0, 100.0) | 158 | 0 (0.0, 100.0) |
|  |  | Week 25 | 150 | 0 (0.0, 66.7) | 154 | 0 (0.0, 100.0) |
|  |  | FU 1 (12 months) | 142 | 0 (0.0, 66.7) | 146 | 0 (0.0, 100.0) |
|  |  | FU 2 (24 months) | 132 | 0 (0.0, 66.7) | 136 | 0 (0.0, 66.7) |
| Insomnia | SL | Baseline | 157 | 33 (0.0, 100.0) | 161 | 33 (0.0, 100.0) |
|  |  | Week 13 | 151 | 33 (0.0, 100.0) | 157 | 33 (0.0, 100.0) |
|  |  | Week 25 | 151 | 33 (0.0, 100.0) | 154 | 33 (0.0, 100.0) |
|  |  | FU 1 (12 months) | 141 | 33 (0.0, 100.0) | 145 | 33 (0.0, 100.0) |
|  |  | FU 2 (24 months) | 133 | 33 (0.0, 100.0) | 136 | 33 (0.0, 100.0) |
| Appetite loss | AP | Baseline | 157 | 0 (0.0, 100.0) | 162 | 0 (0.0, 100.0) |
|  |  | Week 13 | 151 | 0 (0.0, 100.0) | 158 | 0 (0.0, 100.0) |
|  |  | Week 25 | 152 | 0 (0.0, 100.0) | 154 | 0 (0.0, 66.7) |
|  |  | FU 1 (12 months) | 141 | 0 (0.0, 66.7) | 145 | 0 (0.0, 66.7) |
|  |  | FU 2 (24 months) | 133 | 0 (0.0, 66.7) | 136 | 0 (0.0, 100.0) |
| Constipation | CO | Baseline | 157 | 0 (0.0, 100.0) | 162 | 0 (0.0, 100.0) |
|  |  | Week 13 | 151 | 0 (0.0, 100.0) | 158 | 0 (0.0, 100.0) |
|  |  | Week 25 | 152 | 0 (0.0, 100.0) | 154 | 0 (0.0, 100.0) |
|  |  | FU 1 (12 months) | 141 | 0 (0.0, 100.0) | 145 | 0 (0.0, 100.0) |
|  |  | FU 2 (24 months) | 132 | 0 (0.0, 100.0) | 136 | 0 (0.0, 100.0) |
| Diarrhoea | DI | Baseline | 156 | 0 (0.0, 100.0) | 161 | 0 (0.0, 66.7) |
|  |  | Week 13 | 151 | 0 (0.0, 100.0) | 159 | 0 (0.0, 100.0) |
|  |  | Week 25 | 152 | 0 (0.0, 66.7) | 155 | 0 (0.0, 66.7) |
|  |  | FU 1 (12 months) | 142 | 0 (0.0, 100.0) | 145 | 0 (0.0, 100.0) |
|  |  | FU 2 (24 months) | 132 | 0 (0.0, 100.0) | 136 | 0 (0.0, 100.0) |
| Financial difficulties | FI | Baseline | 154 | 0 (0.0, 100.0) | 162 | 0 (0.0, 100.0) |
|  |  | Week 13 | 150 | 0 (0.0, 100.0) | 159 | 0 (0.0, 100.0) |
|  |  | Week 25 | 152 | 0 (0.0, 100.0) | 155 | 0 (0.0, 100.0) |
|  |  | FU 1 (12 months) | 142 | 0 (0.0, 100.0) | 145 | 0 (0.0, 100.0) |
|  |  | FU 2 (24 months) | 132 | 0 (0.0, 100.0) | 136 | 0 (0.0, 100.0) |
| Systemic therapy side effects | BRST | Baseline | 150 | 14 (0.0, 61.9) | 157 | 14 (0.0, 57.1) |
|  |  | Week 13 | 143 | 14 (0.0, 52.4) | 151 | 14 (0.0, 71.4) |
|  |  | Week 25 | 142 | 17 (0.0, 52.4) | 142 | 14 (0.0, 52.4) |
|  |  | FU 1 (12 months) | 133 | 14 (0.0, 66.7) | 140 | 14 (0.0, 57.1) |
|  |  | FU 2 (24 months) | 120 | 19 (0.0, 57.1) | 133 | 14 (0.0, 52.4) |
| Breast symptoms | BRBS | Baseline | 154 | 25 (0.0, 100.0) | 158 | 25 (0.0, 91.7) |
|  |  | Week 13 | 148 | 17 (0.0, 91.7) | 157 | 17 (0.0, 75.0) |
|  |  | Week 25 | 150 | 17 (0.0, 75.0) | 152 | 17 (0.0, 91.7) |
|  |  | FU 1 (12 months) | 141 | 8 (0.0, 75.0) | 144 | 17 (0.0, 75.0) |
|  |  | FU 2 (24 months) | 129 | 8 (0.0, 66.7) | 136 | 8 (0.0, 66.7) |
| Upset by hair loss | BRHL | Baseline | 60 | 33 (0.0, 100.0) | 52 | 33 (0.0, 100.0) |
|  |  | Week 13 | 43 | 0 (0.0, 100.0) | 45 | 0 (0.0, 100.0) |
|  |  | Week 25 | 56 | 0 (0.0, 100.0) | 48 | 17 (0.0, 100.0) |
|  |  | FU 1 (12 months) | 45 | 33 (0.0, 100.0) | 63 | 0 (0.0, 100.0) |
|  |  | FU 2 (24 months) | 53 | 33 (0.0, 100.0) | 58 | 33 (0.0, 100.0) |

Table 14. Mean and SD of the EORTC QLQ-C30 items by treatment arm and visit

|  | | | **Arm A** | | **Arm B** | |
| --- | --- | --- | --- | --- | --- | --- |
| **Item** | **Score** | **Visit** | **N** | **Mean (SD)** | **N** | **Mean (SD)** |
| **Global health status / QoL** | | | | | | |
| Global health status | QL2 | Baseline | 156 | 71 (17.7) | 161 | 72 (19.8) |
|  |  | Week 13 | 151 | 76 (17.8) | 158 | 73 (19.0) |
|  |  | Week 25 | 151 | 75 (17.7) | 155 | 72 (23.3) |
|  |  | FU 1 (12 months) | 142 | 75 (18.8) | 147 | 75 (19.3) |
|  |  | FU 2 (24 months) | 134 | 74 (17.6) | 137 | 73 (22.0) |
| **Functional scales** | | | | | | |
| Physical functioning | PF2 | Baseline | 156 | 91 (10.4) | 161 | 90 (14.0) |
|  |  | Week 13 | 149 | 89 (12.1) | 156 | 87 (16.6) |
|  |  | Week 25 | 146 | 89 (14.1) | 151 | 88 (15.3) |
|  |  | FU 1 (12 months) | 142 | 88 (14.5) | 146 | 86 (16.4) |
|  |  | FU 2 (24 months) | 129 | 88 (14.3) | 133 | 87 (16.5) |
| Role functioning | RF2 | Baseline | 158 | 84 (22.1) | 161 | 80 (26.3) |
|  |  | Week 13 | 150 | 88 (18.5) | 158 | 84 (22.7) |
|  |  | Week 25 | 151 | 84 (21.8) | 154 | 84 (22.1) |
|  |  | FU 1 (12 months) | 142 | 81 (21.8) | 147 | 81 (23.5) |
|  |  | FU 2 (24 months) | 132 | 82 (22.7) | 136 | 83 (22.4) |
| Emotional functioning | EF | Baseline | 155 | 79 (19.5) | 158 | 78 (19.0) |
|  |  | Week 13 | 150 | 83 (18.8) | 157 | 79 (21.5) |
|  |  | Week 25 | 149 | 81 (18.3) | 153 | 81 (21.8) |
|  |  | FU 1 (12 months) | 142 | 80 (21.0) | 144 | 82 (19.1) |
|  |  | FU 2 (24 months) | 131 | 82 (16.9) | 133 | 80 (21.0) |
| Cognitive functioning | CF | Baseline | 153 | 88 (16.2) | 161 | 87 (19.6) |
|  |  | Week 13 | 151 | 86 (19.4) | 157 | 87 (17.6) |
|  |  | Week 25 | 149 | 86 (18.1) | 155 | 88 (16.4) |
|  |  | FU 1 (12 months) | 141 | 86 (19.1) | 143 | 86 (19.4) |
|  |  | FU 2 (24 months) | 133 | 85 (17.9) | 135 | 85 (19.3) |
| Social functioning | SF | Baseline | 155 | 84 (22.0) | 162 | 82 (21.7) |
|  |  | Week 13 | 150 | 92 (15.3) | 157 | 88 (21.4) |
|  |  | Week 25 | 151 | 92 (17.9) | 154 | 90 (18.8) |
|  |  | FU 1 (12 months) | 142 | 91 (15.9) | 144 | 91 (16.0) |
|  |  | FU 2 (24 months) | 131 | 89 (16.9) | 137 | 89 (16.9) |
| Body image | BRBI | Baseline | 155 | 81 (21.5) | 160 | 79 (25.4) |
|  |  | Week 13 | 150 | 85 (21.0) | 155 | 84 (25.1) |
|  |  | Week 25 | 149 | 84 (20.9) | 153 | 86 (21.1) |
|  |  | FU 1 (12 months) | 140 | 84 (21.4) | 144 | 87 (20.4) |
|  |  | FU 2 (24 months) | 131 | 83 (19.9) | 135 | 86 (20.4) |
| Emotional functioning | BRSEF | Baseline | 153 | 76 (25.6) | 154 | 76 (24.9) |
|  |  | Week 13 | 146 | 72 (25.6) | 146 | 76 (26.5) |
|  |  | Week 25 | 141 | 71 (26.1) | 141 | 75 (25.9) |
|  |  | FU 1 (12 months) | 133 | 72 (25.2) | 133 | 75 (24.5) |
|  |  | FU 2 (24 months) | 122 | 73 (24.6) | 121 | 76 (25.4) |
| Sexual enjoyment | BRSEE | Baseline | 69 | 45 (30.8) | 64 | 42 (30.3) |
|  |  | Week 13 | 82 | 43 (29.9) | 76 | 50 (32.0) |
|  |  | Week 25 | 80 | 42 (28.9) | 69 | 41 (31.8) |
|  |  | FU 1 (12 months) | 78 | 44 (28.1) | 63 | 43 (29.0) |
|  |  | FU 2 (24 months) | 70 | 40 (29.3) | 54 | 42 (29.1) |
| Future perspective | BRFU | Baseline | 156 | 56 (29.3) | 159 | 55 (30.8) |
|  |  | Week 13 | 150 | 71 (27.5) | 158 | 66 (28.0) |
|  |  | Week 25 | 151 | 67 (27.6) | 153 | 68 (28.1) |
|  |  | FU 1 (12 months) | 142 | 69 (27.9) | 144 | 70 (29.6) |
|  |  | FU 2 (24 months) | 134 | 72 (24.6) | 136 | 72 (27.0) |
| **Symptom scales / items** | | | | | | |
| Fatigue | FA | Baseline | 155 | 27 (20.5) | 162 | 30 (25.3) |
|  |  | Week 13 | 151 | 24 (20.0) | 157 | 27 (24.6) |
|  |  | Week 25 | 150 | 26 (23.5) | 152 | 26 (24.7) |
|  |  | FU 1 (12 months) | 141 | 25 (19.7) | 143 | 27 (24.0) |
|  |  | FU 2 (24 months) | 131 | 25 (20.0) | 135 | 27 (24.0) |
| Nausea and vomiting | NV | Baseline | 157 | 3 (7.3) | 162 | 2 (7.4) |
|  |  | Week 13 | 151 | 3 (10.7) | 158 | 4 (9.3) |
|  |  | Week 25 | 152 | 2 (6.8) | 154 | 3 (8.0) |
|  |  | FU 1 (12 months) | 141 | 2 (8.2) | 145 | 3 (9.0) |
|  |  | FU 2 (24 months) | 132 | 4 (9.4) | 136 | 3 (8.2) |
| Pain | PA | Baseline | 157 | 15 (18.2) | 160 | 19 (24.7) |
|  |  | Week 13 | 150 | 19 (20.1) | 156 | 23 (25.0) |
|  |  | Week 25 | 152 | 25 (23.7) | 152 | 25 (24.6) |
|  |  | FU 1 (12 months) | 140 | 27 (21.7) | 144 | 27 (26.9) |
|  |  | FU 2 (24 months) | 131 | 27 (23.0) | 135 | 22 (24.2) |
| Dyspnoea | DY | Baseline | 157 | 16 (21.5) | 162 | 16 (23.9) |
|  |  | Week 13 | 151 | 14 (20.9) | 158 | 11 (20.5) |
|  |  | Week 25 | 150 | 10 (19.0) | 154 | 14 (24.0) |
|  |  | FU 1 (12 months) | 142 | 13 (20.7) | 146 | 16 (24.2) |
|  |  | FU 2 (24 months) | 132 | 15 (21.6) | 136 | 12 (19.2) |
| Insomnia | SL | Baseline | 157 | 26 (27.0) | 161 | 28 (31.2) |
|  |  | Week 13 | 151 | 32 (31.3) | 157 | 32 (33.5) |
|  |  | Week 25 | 151 | 31 (29.6) | 154 | 29 (31.5) |
|  |  | FU 1 (12 months) | 141 | 34 (29.9) | 145 | 33 (29.8) |
|  |  | FU 2 (24 months) | 133 | 35 (31.4) | 136 | 29 (29.6) |
| Appetite loss | AP | Baseline | 157 | 7 (17.8) | 162 | 6 (19.5) |
|  |  | Week 13 | 151 | 4 (13.7) | 158 | 6 (15.4) |
|  |  | Week 25 | 152 | 6 (15.9) | 154 | 4 (11.8) |
|  |  | FU 1 (12 months) | 141 | 4 (11.8) | 145 | 6 (14.7) |
|  |  | FU 2 (24 months) | 133 | 4 (13.6) | 136 | 5 (16.8) |
| Constipation | CO | Baseline | 157 | 10 (21.6) | 162 | 8 (19.1) |
|  |  | Week 13 | 151 | 12 (21.5) | 158 | 9 (20.3) |
|  |  | Week 25 | 152 | 10 (21.2) | 154 | 8 (18.8) |
|  |  | FU 1 (12 months) | 141 | 12 (23.0) | 145 | 11 (19.9) |
|  |  | FU 2 (24 months) | 132 | 13 (22.8) | 136 | 10 (20.5) |
| Diarrhoea | DI | Baseline | 156 | 9 (21.3) | 161 | 6 (15.6) |
|  |  | Week 13 | 151 | 7 (16.3) | 159 | 9 (21.6) |
|  |  | Week 25 | 152 | 5 (14.1) | 155 | 3 (10.3) |
|  |  | FU 1 (12 months) | 142 | 5 (15.5) | 145 | 7 (17.3) |
|  |  | FU 2 (24 months) | 132 | 8 (18.9) | 136 | 6 (16.0) |
| Financial difficulties | FI | Baseline | 154 | 8 (18.2) | 162 | 8 (18.4) |
|  |  | Week 13 | 150 | 6 (16.1) | 159 | 8 (19.7) |
|  |  | Week 25 | 152 | 6 (14.7) | 155 | 8 (20.4) |
|  |  | FU 1 (12 months) | 142 | 7 (18.7) | 145 | 6 (17.1) |
|  |  | FU 2 (24 months) | 132 | 5 (16.7) | 136 | 7 (18.5) |
| Systemic therapy side effects | BRST | Baseline | 150 | 17 (13.3) | 157 | 18 (15.1) |
|  |  | Week 13 | 143 | 17 (11.7) | 151 | 18 (14.1) |
|  |  | Week 25 | 142 | 20 (13.8) | 142 | 18 (12.0) |
|  |  | FU 1 (12 months) | 133 | 18 (13.2) | 140 | 18 (12.5) |
|  |  | FU 2 (24 months) | 120 | 19 (14.0) | 133 | 18 (12.3) |
| Breast symptoms | BRBS | Baseline | 154 | 25 (19.0) | 158 | 26 (19.4) |
|  |  | Week 13 | 148 | 19 (17.1) | 157 | 19 (17.2) |
|  |  | Week 25 | 150 | 17 (16.6) | 152 | 19 (19.1) |
|  |  | FU 1 (12 months) | 141 | 15 (17.5) | 144 | 16 (16.0) |
|  |  | FU 2 (24 months) | 129 | 14 (14.8) | 136 | 12 (14.2) |
| Upset by hair loss | BRHL | Baseline | 60 | 34 (35.0) | 52 | 28 (33.3) |
|  |  | Week 13 | 43 | 15 (25.5) | 45 | 18 (24.2) |
|  |  | Week 25 | 56 | 24 (31.6) | 48 | 20 (23.6) |
|  |  | FU 1 (12 months) | 45 | 28 (28.4) | 63 | 21 (28.9) |
|  |  | FU 2 (24 months) | 53 | 34 (34.3) | 58 | 27 (31.5) |

Table 15. Mean change from baseline and 95%CI of the EORTC QLQ-C30 items by treatment arm and visit

|  | | | **Arm A** | | **Arm B** | |
| --- | --- | --- | --- | --- | --- | --- |
| **Item** | **Score** | **Visit** | **N** | **Mean change from baseline (95% CI)** | **N** | **Mean change from baseline (95% CI)** |
| **Global health status / QoL** | | | | | | |
| Global health status | QL2 | Baseline | 156 | 71.4 (68.6, 74.2) | 161 | 72.2 (69.1, 75.3) |
|  |  | Week 13 | 151 | 4.5 (1.4, 7.6) | 158 | 1.6 (-1.9, 5.1) |
|  |  | Week 25 | 151 | 3.6 (0.4, 6.8) | 155 | 0.1 (-4.2, 4.3) |
|  |  | FU 1 (12 months) | 142 | 2.5 (-1.1, 6.1) | 147 | 3.6 (-0.1, 7.2) |
|  |  | FU 2 (24 months) | 134 | 1.4 (-2.4, 5.2) | 137 | 0.7 (-3.5, 4.9) |
| **Functional scales** | | | | | | |
| Physical functioning | PF2 | Baseline | 156 | 91.2 (89.5, 92.8) | 161 | 89.6 (87.4, 91.8) |
|  |  | Week 13 | 149 | -2.1 (-4.0, -0.3) | 156 | -3.0 (-5.3, -0.7) |
|  |  | Week 25 | 146 | -2.4 (-4.5, -0.2) | 151 | -2.0 (-4.2, 0.1) |
|  |  | FU 1 (12 months) | 142 | -4.0 (-6.6, -1.4) | 146 | -3.6 (-6.1, -1.0) |
|  |  | FU 2 (24 months) | 129 | -3.9 (-6.3, -1.4) | 133 | -3.0 (-5.4, -0.6) |
| Role functioning | RF2 | Baseline | 158 | 83.9 (80.4, 87.3) | 161 | 79.7 (75.6, 83.8) |
|  |  | Week 13 | 150 | 3.7 (-0.4, 7.7) | 158 | 3.4 (-1.3, 8.1) |
|  |  | Week 25 | 151 | -0.1 (-4.6, 4.3) | 154 | 3.9 (-0.9, 8.7) |
|  |  | FU 1 (12 months) | 142 | -3.4 (-8.2, 1.4) | 147 | 1.7 (-3.5, 6.9) |
|  |  | FU 2 (24 months) | 132 | -2.3 (-7.4, 2.8) | 136 | 2.2 (-3.3, 7.7) |
| Emotional functioning | EF | Baseline | 155 | 79.0 (75.9, 82.1) | 158 | 77.8 (74.8, 80.8) |
|  |  | Week 13 | 150 | 3.6 (0.5, 6.6) | 157 | 1.2 (-2.6, 5.1) |
|  |  | Week 25 | 149 | 2.6 (-0.5, 5.7) | 153 | 3.4 (-0.3, 7.2) |
|  |  | FU 1 (12 months) | 142 | 0.8 (-3.0, 4.5) | 144 | 4.2 (0.6, 7.8) |
|  |  | FU 2 (24 months) | 131 | 2.2 (-1.3, 5.6) | 133 | 2.5 (-1.6, 6.6) |
| Cognitive functioning | CF | Baseline | 153 | 87.9 (85.3, 90.5) | 161 | 87.3 (84.2, 90.3) |
|  |  | Week 13 | 151 | -2.6 (-5.7, 0.4) | 157 | -0.5 (-3.5, 2.5) |
|  |  | Week 25 | 149 | -2.5 (-5.8, 0.9) | 155 | 0.2 (-2.5, 2.9) |
|  |  | FU 1 (12 months) | 141 | -2.5 (-6.1, 1.2) | 143 | -1.6 (-5.0, 1.8) |
|  |  | FU 2 (24 months) | 133 | -3.3 (-6.5, 0.0) | 135 | -2.8 (-6.4, 0.7) |
| Social functioning | SF | Baseline | 155 | 83.7 (80.2, 87.2) | 162 | 82.3 (78.9, 85.7) |
|  |  | Week 13 | 150 | 7.3 (4.2, 10.5) | 157 | 5.6 (1.9, 9.3) |
|  |  | Week 25 | 151 | 7.5 (3.7, 11.3) | 154 | 7.7 (3.9, 11.5) |
|  |  | FU 1 (12 months) | 142 | 6.0 (2.1, 9.9) | 144 | 9.5 (5.7, 13.3) |
|  |  | FU 2 (24 months) | 131 | 5.9 (1.3, 10.4) | 137 | 6.8 (2.8, 10.8) |
| Body image | BRBI | Baseline | 155 | 81.0 (77.6, 84.4) | 160 | 79.2 (75.2, 83.1) |
|  |  | Week 13 | 150 | 3.8 (1.0, 6.7) | 155 | 3.8 (0.7, 6.9) |
|  |  | Week 25 | 149 | 3.0 (-0.2, 6.1) | 153 | 5.6 (2.4, 8.8) |
|  |  | FU 1 (12 months) | 140 | 3.6 (0.2, 7.0) | 144 | 6.7 (2.9, 10.4) |
|  |  | FU 2 (24 months) | 131 | 3.0 (-0.6, 6.6) | 135 | 5.8 (1.6, 10.0) |
| Emotional functioning | BRSEF | Baseline | 153 | 76.3 (72.2, 80.3) | 154 | 76.3 (72.3, 80.3) |
|  |  | Week 13 | 146 | -3.4 (-6.9, 0.0) | 146 | -0.7 (-4.6, 3.3) |
|  |  | Week 25 | 141 | -4.3 (-7.8, -0.7) | 141 | -1.7 (-5.5, 2.2) |
|  |  | FU 1 (12 months) | 133 | -3.1 (-7.0, 0.8) | 133 | -1.8 (-6.3, 2.8) |
|  |  | FU 2 (24 months) | 122 | -1.8 (-5.9, 2.3) | 121 | -2.2 (-7.0, 2.6) |
| Sexual enjoyment | BRSEE | Baseline | 69 | 45.4 (38.0, 52.8) | 64 | 41.7 (34.1, 49.2) |
|  |  | Week 13 | 82 | -0.8 (-8.2, 6.6) | 76 | 12.3 (4.8, 19.7) |
|  |  | Week 25 | 80 | -1.7 (-9.2, 5.9) | 69 | 4.8 (-3.1, 12.8) |
|  |  | FU 1 (12 months) | 78 | -0.9 (-8.4, 6.7) | 63 | 3.7 (-5.0, 12.4) |
|  |  | FU 2 (24 months) | 70 | -6.7 (-15.4, 2.1) | 54 | 3.1 (-7.0, 13.1) |
| Future perspective | BRFU | Baseline | 156 | 56.2 (51.6, 60.8) | 159 | 54.5 (49.7, 59.3) |
|  |  | Week 13 | 150 | 14.7 (9.9, 19.5) | 158 | 12.9 (8.2, 17.6) |
|  |  | Week 25 | 151 | 10.8 (6.1, 15.6) | 153 | 13.7 (8.4, 19.1) |
|  |  | FU 1 (12 months) | 142 | 13.4 (8.4, 18.4) | 144 | 15.7 (10.4, 21.1) |
|  |  | FU 2 (24 months) | 134 | 16.2 (11.2, 21.1) | 136 | 19.1 (13.7, 24.5) |
| **Symptom scales / items** | | | | | | |
| Fatigue | FA | Baseline | 155 | 26.9 (23.6, 30.1) | 162 | 30.0 (26.1, 34.0) |
|  |  | Week 13 | 151 | -2.3 (-5.8, 1.2) | 157 | -3.0 (-7.3, 1.2) |
|  |  | Week 25 | 150 | -0.7 (-5.1, 3.6) | 152 | -4.1 (-8.3, 0.1) |
|  |  | FU 1 (12 months) | 141 | -2.1 (-6.3, 2.0) | 143 | -2.8 (-7.0, 1.4) |
|  |  | FU 2 (24 months) | 131 | -1.7 (-6.2, 2.9) | 135 | -2.8 (-7.4, 1.8) |
| Nausea and vomiting | NV | Baseline | 157 | 2.8 (1.6, 3.9) | 162 | 2.4 (1.2, 3.5) |
|  |  | Week 13 | 151 | 0.1 (-1.6, 1.8) | 158 | 1.2 (-0.6, 2.9) |
|  |  | Week 25 | 152 | -0.4 (-1.7, 0.8) | 154 | 0.3 (-1.3, 1.9) |
|  |  | FU 1 (12 months) | 141 | -0.2 (-2.0, 1.5) | 145 | 0.6 (-1.2, 2.3) |
|  |  | FU 2 (24 months) | 132 | 0.9 (-1.0, 2.7) | 136 | 0.5 (-1.0, 2.0) |
| Pain | PA | Baseline | 157 | 15.1 (12.2, 17.9) | 160 | 18.5 (14.7, 22.4) |
|  |  | Week 13 | 150 | 4.3 (1.1, 7.6) | 156 | 4.4 (-0.1, 8.9) |
|  |  | Week 25 | 152 | 9.3 (5.5, 13.1) | 152 | 6.2 (1.5, 11.0) |
|  |  | FU 1 (12 months) | 140 | 11.4 (7.4, 15.4) | 144 | 8.4 (3.6, 13.3) |
|  |  | FU 2 (24 months) | 131 | 12.0 (7.1, 16.8) | 135 | 4.8 (0.1, 9.5) |
| Dyspnoea | DY | Baseline | 157 | 15.7 (12.3, 19.1) | 162 | 16.5 (12.8, 20.2) |
|  |  | Week 13 | 151 | -1.3 (-5.6, 2.9) | 158 | -4.6 (-8.5, -0.7) |
|  |  | Week 25 | 150 | -4.9 (-9.0, -0.8) | 154 | -1.9 (-6.4, 2.5) |
|  |  | FU 1 (12 months) | 142 | -1.9 (-6.1, 2.3) | 146 | 0.5 (-3.8, 4.7) |
|  |  | FU 2 (24 months) | 132 | 0.3 (-4.3, 4.8) | 136 | -4.2 (-8.6, 0.3) |
| Insomnia | SL | Baseline | 157 | 26.3 (22.1, 30.6) | 161 | 28.4 (23.5, 33.2) |
|  |  | Week 13 | 151 | 5.7 (1.2, 10.3) | 157 | 3.8 (-1.4, 9.1) |
|  |  | Week 25 | 151 | 4.6 (-0.2, 9.5) | 154 | 0.2 (-5.2, 5.6) |
|  |  | FU 1 (12 months) | 141 | 7.6 (2.5, 12.7) | 145 | 5.5 (0.0, 11.0) |
|  |  | FU 2 (24 months) | 133 | 9.3 (4.1, 14.5) | 136 | 1.5 (-4.4, 7.3) |
| Appetite loss | AP | Baseline | 157 | 7.2 (4.4, 10.0) | 162 | 6.4 (3.4, 9.4) |
|  |  | Week 13 | 151 | -2.6 (-5.4, 0.1) | 158 | -0.4 (-4.2, 3.4) |
|  |  | Week 25 | 152 | -0.9 (-4.2, 2.4) | 154 | -3.0 (-6.2, 0.1) |
|  |  | FU 1 (12 months) | 141 | -3.3 (-6.1, -0.5) | 145 | -0.7 (-4.1, 2.7) |
|  |  | FU 2 (24 months) | 133 | -3.0 (-5.9, -0.1) | 136 | -0.2 (-3.9, 3.4) |
| Constipation | CO | Baseline | 157 | 10.4 (7.0, 13.8) | 162 | 8.4 (5.5, 11.4) |
|  |  | Week 13 | 151 | 1.3 (-2.1, 4.8) | 158 | 0.8 (-2.5, 4.2) |
|  |  | Week 25 | 152 | -1.1 (-5.1, 2.9) | 154 | -0.2 (-3.3, 2.9) |
|  |  | FU 1 (12 months) | 141 | 1.2 (-2.8, 5.2) | 145 | 2.3 (-1.0, 5.6) |
|  |  | FU 2 (24 months) | 132 | 2.0 (-2.5, 6.5) | 136 | 1.5 (-2.2, 5.1) |
| Diarrhoea | DI | Baseline | 156 | 9.2 (5.8, 12.6) | 161 | 6.4 (4.0, 8.8) |
|  |  | Week 13 | 151 | -2.9 (-6.1, 0.4) | 159 | 2.1 (-1.5, 5.7) |
|  |  | Week 25 | 152 | -3.9 (-7.7, -0.2) | 155 | -3.7 (-6.2, -1.1) |
|  |  | FU 1 (12 months) | 142 | -4.2 (-7.6, -0.9) | 145 | 0.9 (-2.4, 4.3) |
|  |  | FU 2 (24 months) | 132 | -2.0 (-6.0, 1.9) | 136 | 0.0 (-3.0, 3.0) |
| Financial difficulties | FI | Baseline | 154 | 7.8 (4.9, 10.7) | 162 | 7.8 (5.0, 10.7) |
|  |  | Week 13 | 150 | -1.1 (-4.0, 1.8) | 159 | 0.0 (-2.9, 2.9) |
|  |  | Week 25 | 152 | -1.3 (-4.4, 1.8) | 155 | 0.0 (-3.2, 3.2) |
|  |  | FU 1 (12 months) | 142 | -0.9 (-4.0, 2.1) | 145 | -2.1 (-5.2, 1.0) |
|  |  | FU 2 (24 months) | 132 | -2.8 (-6.3, 0.7) | 136 | -1.0 (-4.1, 2.1) |
| Systemic therapy side effects | BRST | Baseline | 150 | 17.4 (15.2, 19.5) | 157 | 18.4 (16.0, 20.7) |
|  |  | Week 13 | 143 | 0.1 (-2.3, 2.5) | 151 | -0.8 (-3.4, 1.9) |
|  |  | Week 25 | 142 | 2.3 (-0.4, 5.0) | 142 | 0.1 (-2.6, 2.8) |
|  |  | FU 1 (12 months) | 133 | 0.9 (-2.0, 3.7) | 140 | 0.0 (-2.7, 2.8) |
|  |  | FU 2 (24 months) | 120 | 2.3 (-0.8, 5.4) | 133 | -0.6 (-3.5, 2.2) |
| Breast symptoms | BRBS | Baseline | 154 | 24.6 (21.6, 27.6) | 158 | 25.9 (22.9, 29.0) |
|  |  | Week 13 | 148 | -6.1 (-9.7, -2.6) | 157 | -6.9 (-9.9, -3.9) |
|  |  | Week 25 | 150 | -8.0 (-11.6, -4.4) | 152 | -7.2 (-10.9, -3.5) |
|  |  | FU 1 (12 months) | 141 | -10.3 (-14.0, -6.6) | 144 | -9.7 (-13.3, -6.0) |
|  |  | FU 2 (24 months) | 129 | -11.0 (-14.9, -7.2) | 136 | -13.1 (-16.5, -9.6) |
| Upset by hair loss | BRHL | Baseline | 60 | 33.9 (24.9, 42.9) | 52 | 28.2 (18.9, 37.5) |
|  |  | Week 13 | 43 | -16.3 (-26.4, -6.2) | 44 | -16.7 (-29.3, -4.0) |
|  |  | Week 25 | 56 | -6.5 (-18.0, 4.9) | 47 | -12.1 (-24.4, 0.3) |
|  |  | FU 1 (12 months) | 45 | -3.7 (-13.3, 5.9) | 62 | -10.2 (-21.5, 1.1) |
|  |  | FU 2 (24 months) | 53 | -5.7 (-17.6, 6.3) | 57 | -8.2 (-21.1, 4.7) |

Table 16. Results from a nonparametric rank based model for the Quality of life items

| **Item** | **Scale** | **Effect** | **ANOVA-Type Statistic** | **P-value** |
| --- | --- | --- | --- | --- |
| Global health status | QL2 | Arm | 0.044 | 0.833 |
|  |  | Time | 3.402 | 0.011 |
|  |  | Arm:Time | 1.479 | 0.211 |
| Physical functioning | PF2 | Arm | 0.055 | 0.814 |
|  |  | Time | 4.463 | 0.002 |
|  |  | Arm:Time | 0.062 | 0.99 |
| Role functioning | RF2 | Arm | 0.139 | 0.709 |
|  |  | Time | 2.841 | 0.028 |
|  |  | Arm:Time | 1.193 | 0.312 |
| Emotional functioning | EF | Arm | 0.152 | 0.697 |
|  |  | Time | 3.555 | 0.008 |
|  |  | Arm:Time | 1.579 | 0.18 |
| Cognitive functioning | CF | Arm | 0.176 | 0.675 |
|  |  | Time | 1.633 | 0.164 |
|  |  | Arm:Time | 0.209 | 0.931 |
| Social functioning | SF | Arm | 0.403 | 0.526 |
|  |  | Time | 16.130 | <0.001 |
|  |  | Arm:Time | 0.921 | 0.444 |
| Fatigue | FA | Arm | 0.125 | 0.723 |
|  |  | Time | 2.164 | 0.077 |
|  |  | Arm:Time | 0.332 | 0.839 |
| Nausea and vomiting | NV | Arm | 0.002 | 0.963 |
|  |  | Time | 0.260 | 0.896 |
|  |  | Arm:Time | 0.949 | 0.431 |
| Pain | PA | Arm | 0.346 | 0.556 |
|  |  | Time | 16.428 | <0.001 |
|  |  | Arm:Time | 2.543 | 0.041 |
| Dyspnoea | DY | Arm | 0.148 | 0.7 |
|  |  | Time | 2.821 | 0.026 |
|  |  | Arm:Time | 2.300 | 0.06 |
| Insomnia | SL | Arm | 0.611 | 0.434 |
|  |  | Time | 3.669 | 0.006 |
|  |  | Arm:Time | 0.705 | 0.583 |
| Appetite loss | AP | Arm | 0.023 | 0.878 |
|  |  | Time | 1.061 | 0.373 |
|  |  | Arm:Time | 2.193 | 0.069 |
| Constipation | CO | Arm | 0.915 | 0.339 |
|  |  | Time | 2.172 | 0.073 |
|  |  | Arm:Time | 0.238 | 0.909 |
| Diarrhoea | DI | Arm | 0.127 | 0.721 |
|  |  | Time | 2.396 | 0.05 |
|  |  | Arm:Time | 1.778 | 0.133 |
| Financial difficulties | FI | Arm | 0.165 | 0.685 |
|  |  | Time | 2.868 | 0.028 |
|  |  | Arm:Time | 0.281 | 0.866 |
| Body image | BRBI | Arm | 1.481 | 0.224 |
|  |  | Time | 9.061 | <0.001 |
|  |  | Arm:Time | 1.337 | 0.257 |
| Emotional functioning | BRSEF | Arm | 1.507 | 0.22 |
|  |  | Time | 2.021 | 0.098 |
|  |  | Arm:Time | 0.963 | 0.419 |
| Sexual enjoyment | BRSEE | Arm | 0.036 | 0.85 |
|  |  | Time | 1.252 | 0.287 |
|  |  | Arm:Time | 1.027 | 0.388 |
| Future perspective | BRFU | Arm | 0.057 | 0.812 |
|  |  | Time | 28.571 | <0.001 |
|  |  | Arm:Time | 1.073 | 0.368 |
| Systemic therapy side effects | BRST | Arm | 0.324 | 0.569 |
|  |  | Time | 1.462 | 0.216 |
|  |  | Arm:Time | 0.248 | 0.893 |
| Breast symptoms | BRBS | Arm | 0.003 | 0.955 |
|  |  | Time | 35.827 | <0.001 |
|  |  | Arm:Time | 0.044 | 0.833 |
| Upset by hair loss | BRHL | Arm | 3.402 | 0.011 |
|  |  | Time | 1.479 | 0.211 |
|  |  | Arm:Time | 0.055 | 0.814 |


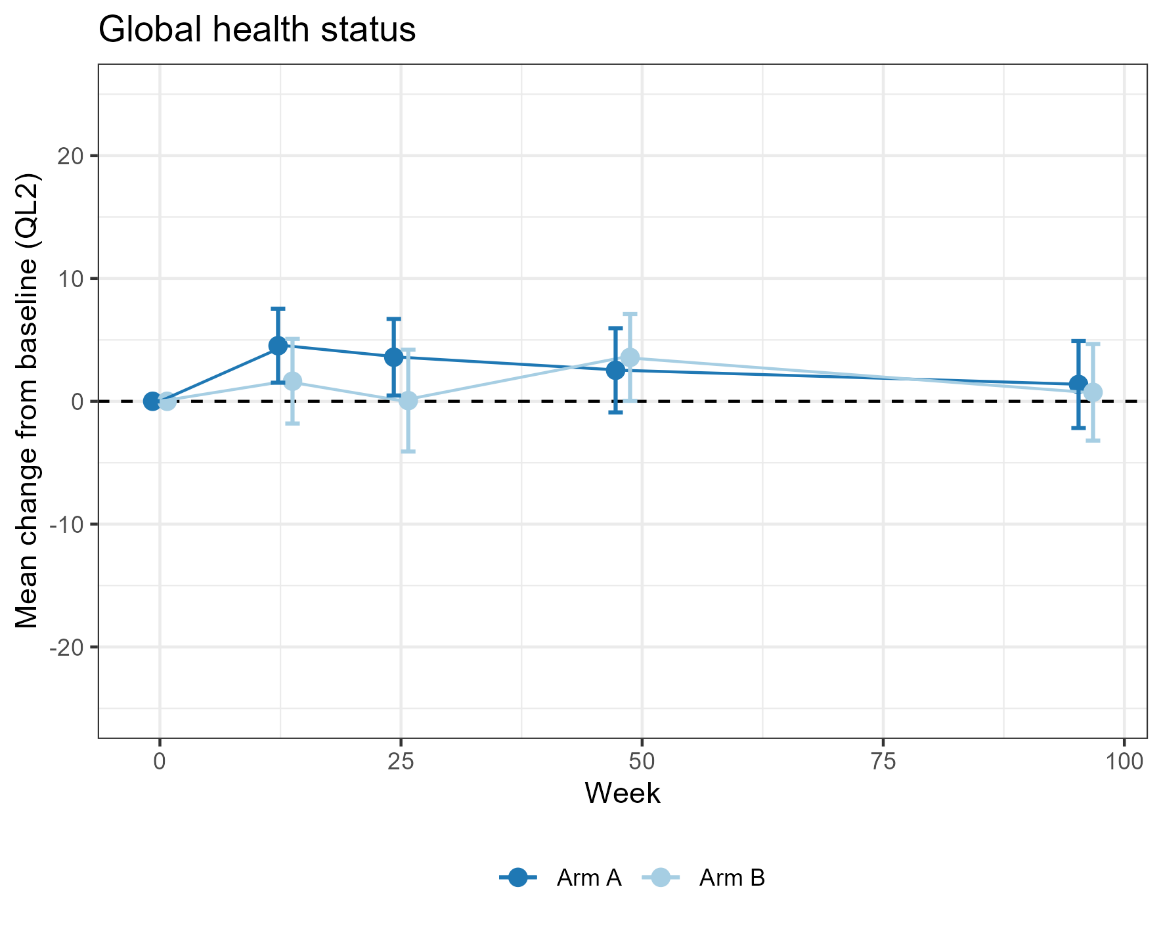


Figure 4. Mean change from baseline and associated 95% CI for Global health status by arm and week


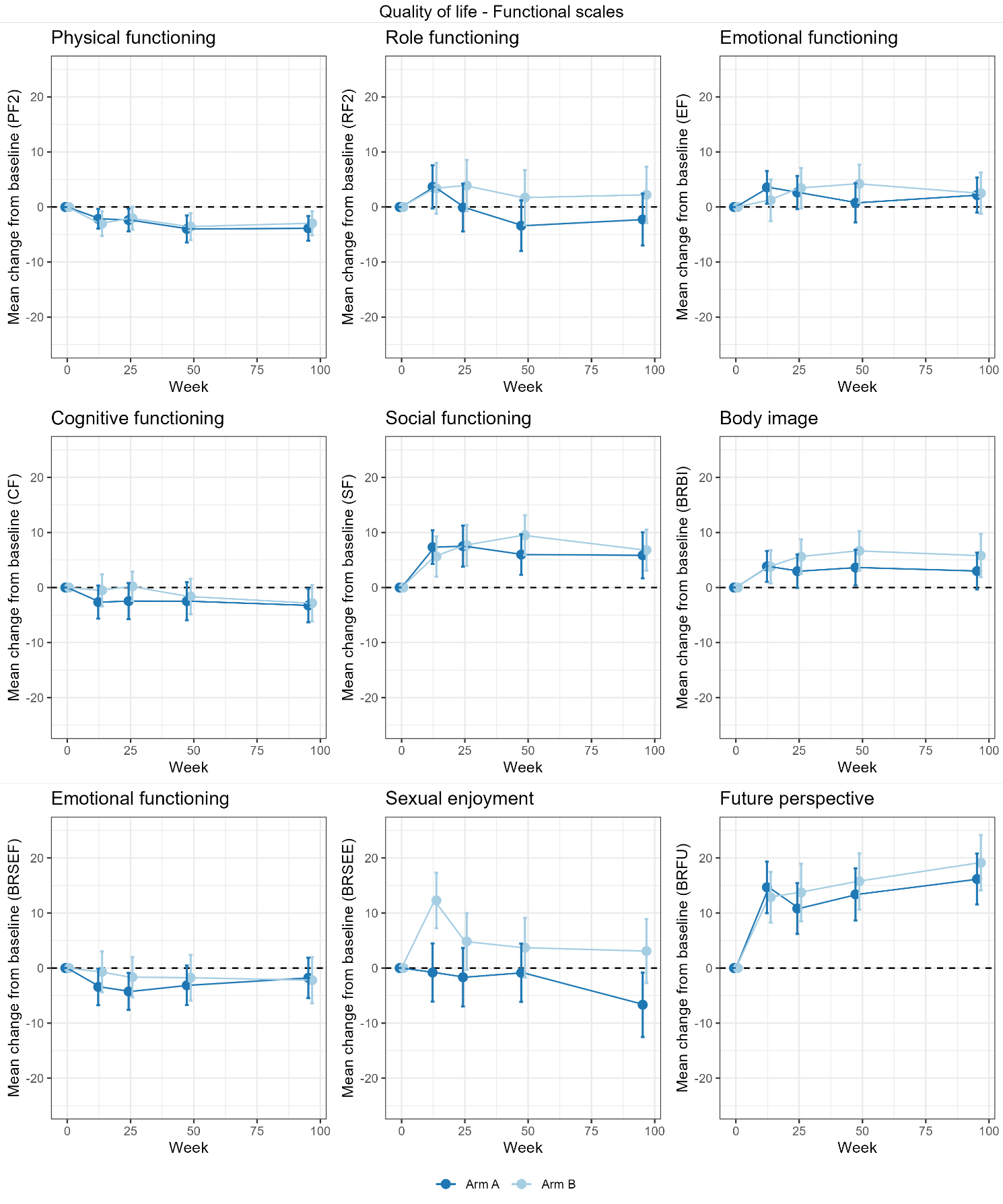


Figure 5. Mean change from baseline and associated 95% CI for functional Quality of life items by arm and week.


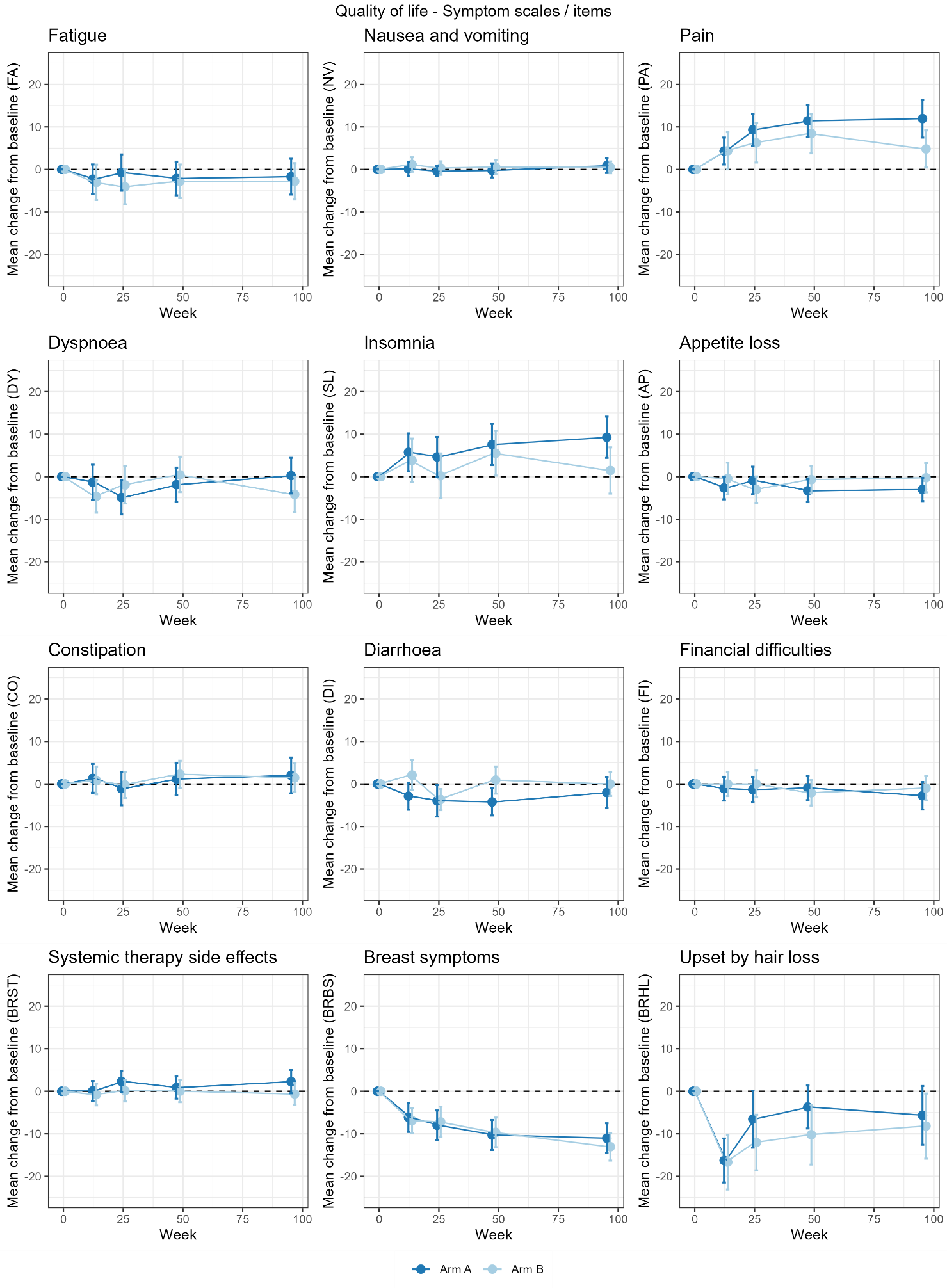


Figure 6. Mean change from baseline and associated 95% CI for symptom Quality of life scales / items by arm and week.

#### Intensity of muscle or joint pain/stiffness and its impact on everyday functioning

Severity of muscle or joint pain/stiffness was not only measured by the single-item "worst pain" (see primary endpoint) but also by the four pain severity items. A composite of the four pain items, a mean severity score, was calculated. In addition, the BPI-SF [13] measures how much pain has interfered with seven daily activities, including general activity, walking, work, mood, enjoyment of life, relations with others, and sleep. Pain interference was calculated as the mean of the seven interference items. This BPI-SF was completed every 3 weeks during the intervention phase and during the follow-up phase at 12 and 24 months (± 61 days). The median and range for Mean BPI-SF and Mean severity score by treatment arm and visit are shown in Table 17, mean and SD in Table 18 and mean change from baseline and associated 95% CI in Table 19.

The Mean severity score and mean BPI-SF were analyzed using a nonparametric rank-based model for longitudinal data [3]. There was no significant difference between treatment arms, but there was a significant effect of time (Table 20, Figure 7).

Table 17. Median and range of the Mean severity scores by treatment arm and visit

|  | | | **Arm A** | | **Arm B** | |
| --- | --- | --- | --- | --- | --- | --- |
| **Endpoint** | **Score** | **Visit** | **N** | **Median (range)** | **N** | **Median (range)** |
| Intensity of muscle or joint pain/stiffness and its impact on everyday functioning | Mean BPI-SF | Eligibility | 158 | 0 (0.0, 3.3) | 162 | 0 (0.0, 6.9) |
|  |  | Week 4 | 154 | 0 (0.0, 6.0) | 160 | 0 (0.0, 6.1) |
|  |  | Week 7 | 156 | 0 (0.0, 7.3) | 160 | 0 (0.0, 6.4) |
|  |  | Week 10 | 154 | 1 (0.0, 8.3) | 159 | 0 (0.0, 6.3) |
|  |  | Week 13 | 153 | 1 (0.0, 7.8) | 158 | 0 (0.0, 7.9) |
|  |  | Week 16 | 155 | 1 (0.0, 8.1) | 159 | 1 (0.0, 9.7) |
|  |  | Week 19 | 148 | 0 (0.0, 8.1) | 160 | 1 (0.0, 8.3) |
|  |  | Week 22 | 150 | 1 (0.0, 6.1) | 156 | 1 (0.0, 6.9) |
|  |  | Week 25 | 153 | 1 (0.0, 8.0) | 155 | 1 (0.0, 6.3) |
|  |  | FU 1 (12 months) | 140 | 1 (0.0, 8.0) | 145 | 1 (0.0, 7.7) |
|  |  | FU 2 (24 months) | 134 | 1 (0.0, 7.3) | 136 | 1 (0.0, 7.3) |
|  | Mean severity score | Eligibility | 158 | 1 (0.0, 2.3) | 162 | 0 (0.0, 3.3) |
|  |  | Week 4 | 154 | 1 (0.0, 7.3) | 159 | 1 (0.0, 7.8) |
|  |  | Week 7 | 156 | 1 (0.0, 5.5) | 160 | 1 (0.0, 7.0) |
|  |  | Week 10 | 154 | 1 (0.0, 6.8) | 159 | 1 (0.0, 7.3) |
|  |  | Week 13 | 153 | 1 (0.0, 6.3) | 159 | 1 (0.0, 8.0) |
|  |  | Week 16 | 155 | 1 (0.0, 6.5) | 159 | 1 (0.0, 8.0) |
|  |  | Week 19 | 148 | 1 (0.0, 8.0) | 160 | 1 (0.0, 9.0) |
|  |  | Week 22 | 151 | 1 (0.0, 8.3) | 156 | 1 (0.0, 8.0) |
|  |  | Week 25 | 152 | 1 (0.0, 8.0) | 154 | 2 (0.0, 8.3) |
|  |  | FU 1 (12 months) | 142 | 2 (0.0, 8.0) | 147 | 2 (0.0, 9.0) |
|  |  | FU 2 (24 months) | 134 | 2 (0.0, 6.8) | 137 | 1 (0.0, 6.5) |

Table 18. Mean and SD of the Mean severity scores by treatment arm and visit

|  | | | **Arm A** | | **Arm B** | |
| --- | --- | --- | --- | --- | --- | --- |
| **Endpoint** | **Score** | **Visit** | **N** | **Mean (SD)** | **N** | **Mean (SD)** |
| Intensity of muscle or joint pain/stiffness and its impact on everyday functioning | Mean BPI-SF | Eligibility | 158 | 0.5 (0.73) | 162 | 0.6 (1.18) |
|  |  | Week 4 | 154 | 1.0 (1.37) | 160 | 1.0 (1.38) |
|  |  | Week 7 | 156 | 1.0 (1.45) | 160 | 0.9 (1.26) |
|  |  | Week 10 | 154 | 1.1 (1.50) | 159 | 1.1 (1.38) |
|  |  | Week 13 | 153 | 1.1 (1.42) | 158 | 1.2 (1.75) |
|  |  | Week 16 | 155 | 1.1 (1.47) | 159 | 1.2 (1.59) |
|  |  | Week 19 | 148 | 1.1 (1.57) | 160 | 1.1 (1.59) |
|  |  | Week 22 | 150 | 1.1 (1.38) | 156 | 1.2 (1.54) |
|  |  | Week 25 | 153 | 1.1 (1.45) | 155 | 1.2 (1.59) |
|  |  | FU 1 (12 months) | 140 | 1.6 (1.71) | 145 | 1.5 (1.67) |
|  |  | FU 2 (24 months) | 134 | 1.5 (1.61) | 136 | 1.4 (1.59) |
|  | Mean severity score | Eligibility | 158 | 0.6 (0.66) | 162 | 0.4 (0.58) |
|  |  | Week 4 | 154 | 1.5 (1.56) | 159 | 1.4 (1.61) |
|  |  | Week 7 | 156 | 1.5 (1.40) | 160 | 1.4 (1.51) |
|  |  | Week 10 | 154 | 1.5 (1.56) | 159 | 1.5 (1.69) |
|  |  | Week 13 | 153 | 1.5 (1.32) | 159 | 1.6 (1.80) |
|  |  | Week 16 | 155 | 1.7 (1.53) | 159 | 1.7 (1.78) |
|  |  | Week 19 | 148 | 1.6 (1.58) | 160 | 1.7 (1.77) |
|  |  | Week 22 | 151 | 1.7 (1.64) | 156 | 1.7 (1.79) |
|  |  | Week 25 | 152 | 1.8 (1.75) | 154 | 1.8 (1.81) |
|  |  | FU 1 (12 months) | 142 | 2.3 (1.93) | 147 | 2.2 (1.98) |
|  |  | FU 2 (24 months) | 134 | 2.2 (1.87) | 137 | 1.8 (1.76) |

Table 19. Mean change from baseline and associated 95% CI for Mean severity scores by treatment arm and visit

|  | | | **Arm A** | | **Arm B** | |
| --- | --- | --- | --- | --- | --- | --- |
| **Endpoint** | **Score** | **Visit** | **N** | **Mean change from baseline (95% CI)** | **N** | **Mean change from baseline (95% CI)** |
| Intensity of muscle or joint pain/stiffness and its impact on everyday functioning | Change From Baseline | Eligibility | 158 | 0.0 | 162 | 0.0 |
|  |  | Week 4 | 154 | 0.9 (0.68, 1.19) | 159 | 1.0 (0.74, 1.23) |
|  |  | Week 7 | 156 | 0.9 (0.71, 1.19) | 160 | 1.0 (0.77, 1.24) |
|  |  | Week 10 | 154 | 0.9 (0.66, 1.15) | 159 | 1.1 (0.86, 1.39) |
|  |  | Week 13 | 153 | 0.9 (0.65, 1.08) | 159 | 1.2 (0.94, 1.50) |
|  |  | Week 16 | 155 | 1.1 (0.83, 1.30) | 159 | 1.3 (1.01, 1.57) |
|  |  | Week 19 | 148 | 1.1 (0.80, 1.30) | 160 | 1.3 (1.04, 1.58) |
|  |  | Week 22 | 151 | 1.2 (0.89, 1.41) | 156 | 1.3 (0.99, 1.54) |
|  |  | Week 25 | 152 | 1.2 (0.91, 1.45) | 154 | 1.4 (1.10, 1.68) |
|  |  | FU 1 (12 months) | 142 | 1.7 (1.39, 2.03) | 147 | 1.7 (1.43, 2.06) |
|  |  | FU 2 (24 months) | 134 | 1.6 (1.31, 1.94) | 137 | 1.4 (1.16, 1.73) |
|  |  | Eligibility | 158 | 0.0 | 162 | 0.0 |
|  |  | Week 4 | 154 | 0.5 (0.34, 0.73) | 160 | 0.4 (0.21, 0.63) |
|  |  | Week 7 | 156 | 0.5 (0.33, 0.77) | 160 | 0.3 (0.12, 0.55) |
|  |  | Week 10 | 154 | 0.6 (0.38, 0.83) | 159 | 0.4 (0.23, 0.67) |
|  |  | Week 13 | 153 | 0.6 (0.35, 0.79) | 158 | 0.6 (0.35, 0.92) |
|  |  | Week 16 | 155 | 0.6 (0.37, 0.82) | 159 | 0.6 (0.31, 0.81) |
|  |  | Week 19 | 148 | 0.6 (0.39, 0.90) | 160 | 0.5 (0.28, 0.77) |
|  |  | Week 22 | 150 | 0.6 (0.34, 0.78) | 156 | 0.6 (0.32, 0.79) |
|  |  | Week 25 | 153 | 0.6 (0.38, 0.84) | 155 | 0.6 (0.36, 0.87) |
|  |  | FU 1 (12 months) | 140 | 1.1 (0.83, 1.39) | 145 | 0.9 (0.62, 1.21) |
|  |  | FU 2 (24 months) | 134 | 1.0 (0.71, 1.25) | 136 | 0.8 (0.55, 1.13) |

Table 20. Results from a nonparametric rank based model for the Mean severity score and mean BPI-SF

| **Endpoint** | **Scale** | **Effect** | **ANOVA-Type Statistic** | **P-value** |
| --- | --- | --- | --- | --- |
| Pain severity | Mean severity score | Arm | 1.048 | 0.306 |
|  |  | Time | 47.324 | <0.001 |
|  |  | Arm:Time | 0.825 | 0.571 |
| Impact on everyday functioning | Mean BPI-SF | Arm | 0.344 | 0.557 |
|  |  | Time | 31.584 | <0.001 |
|  |  | Arm:Time | 0.387 | 0.917 |


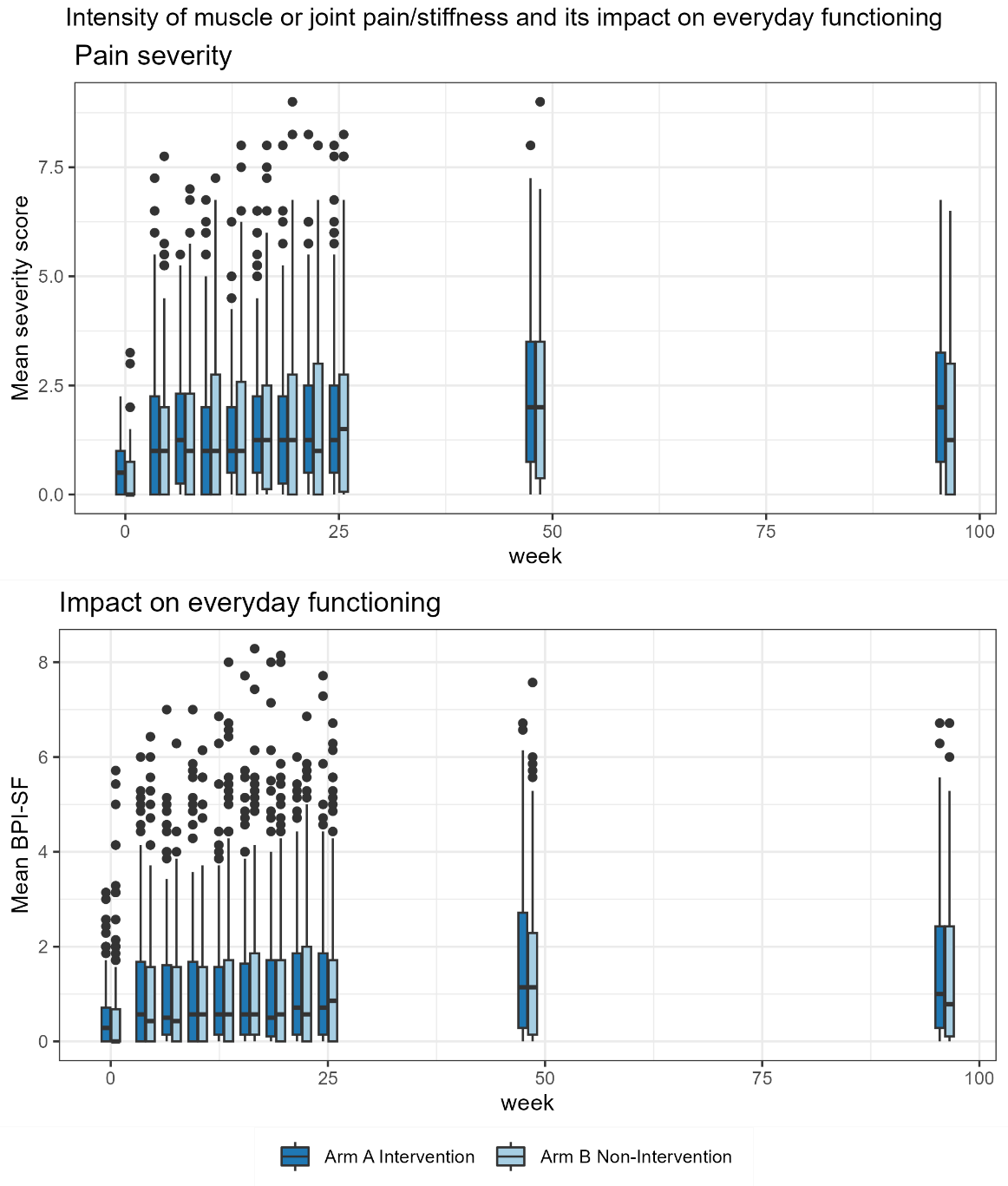


Figure 7. Boxplots of mean severity scores and mean BPI-SF scores by treatment arm and visit

#### Walking activity

During trial intervention phase, the daily number of steps and the number of minutes spent doing activity (e.g. walking) were summarized by week and treatment arm in Table 21 and Table 22. Reasons for not reaching the set activity goal of 30 minutes for at least 5 days per week for patients in arm A (collected by patient diary) are given in **Error! Reference source not found.** and the proportion of patients who did not reach this goal is shown in **Error! Reference source not found.**. Detailed listing of patient who had their judgement regarding to reaching the goal different from according to protocol can be found in the Appendix (**Error! Reference source not found.**). During the follow-up phase at 12 and 24 months (± 61 days), walking habits (collected by questionnaire) were reported and the proportion of patients walking 30 minutes for at least 5 days per week was calculated (Table 23). Physical activity was also described according to employment status (Table 24, Figure 8), smoking status (Table 25, Figure 9) and marital status (Table 26). Finally, mean and median daily number of steps and number of minutes spent doing activity over the whole intervention phase are summarized in Table 27.

Table 21. Median daily number of steps and of minutes spent doing activity (e.g. walking) by week and treatment arm (N= number of patients)

|  | | | **Arm A** | | **Arm B** | |
| --- | --- | --- | --- | --- | --- | --- |
| **Endpoint** | **Measurement** | **Week** | **N** | **Median (range)** | **N** | **Median (range)** |
| Walking activity | Median daily number of steps | 1 | 151 | 7335 (0, 17380) | 155 | 7041 (0, 31510) |
|  |  | 2 | 155 | 9172 (0, 17869) | 158 | 7684 (0, 23864) |
|  |  | 3 | 156 | 9586 (0, 27632) | 159 | 7926 (0, 22187) |
|  |  | 4 | 156 | 8988 (0, 17951) | 161 | 7886 (110, 24057) |
|  |  | 5 | 154 | 8932 (0, 18125) | 159 | 7820 (43, 21670) |
|  |  | 6 | 150 | 8674 (0, 19071) | 158 | 7827 (0, 23251) |
|  |  | 7 | 152 | 8198 (0, 20681) | 159 | 7883 (31, 22616) |
|  |  | 8 | 150 | 8783 (0, 17392) | 158 | 7589 (0, 21365) |
|  |  | 9 | 148 | 8874 (0, 20724) | 158 | 7331 (728, 20154) |
|  |  | 10 | 151 | 8361 (0, 19186) | 157 | 7714 (96, 20251) |
|  |  | 11 | 145 | 8408 (0, 17701) | 156 | 7199 (0, 20216) |
|  |  | 12 | 147 | 8858 (0, 19972) | 157 | 7343 (0, 19565) |
|  |  | 13 | 148 | 8473 (0, 18990) | 157 | 7615 (0, 21122) |
|  |  | 14 | 145 | 8559 (0, 18164) | 154 | 7086 (0, 19814) |
|  |  | 15 | 143 | 8690 (0, 20007) | 156 | 7402 (0, 18983) |
|  |  | 16 | 142 | 8553 (0, 20899) | 155 | 7144 (516, 17296) |
|  |  | 17 | 147 | 8273 (0, 18994) | 155 | 7454 (526, 22399) |
|  |  | 18 | 148 | 8841 (0, 25212) | 156 | 7128 (103, 22222) |
|  |  | 19 | 147 | 8646 (0, 16936) | 156 | 7234 (354, 18178) |
|  |  | 20 | 143 | 8154 (0, 15813) | 152 | 7682 (457, 24815) |
|  |  | 21 | 143 | 8085 (0, 18461) | 151 | 7561 (0, 21110) |
|  |  | 22 | 146 | 7815 (0, 17764) | 148 | 7250 (419, 20279) |
|  |  | 23 | 144 | 7897 (0, 17054) | 146 | 7335 (0, 19135) |
|  |  | 24 | 140 | 7876 (0, 17519) | 140 | 7456 (1087, 20558) |
|  |  | 25 | 125 | 6858 (0, 16598) | 136 | 7036 (0, 20710) |
|  | Median number of minutes spent doing activity | 1 | 151 | 73 (0, 164) | 155 | 75 (0, 272) |
|  |  | 2 | 155 | 87 (0, 161) | 158 | 81 (0, 205) |
|  |  | 3 | 156 | 91 (0, 209) | 159 | 84 (0, 197) |
|  |  | 4 | 156 | 87 (0, 172) | 161 | 84 (1, 217) |
|  |  | 5 | 154 | 86 (0, 173) | 159 | 81 (0, 190) |
|  |  | 6 | 150 | 85 (0, 183) | 158 | 80 (0, 219) |
|  |  | 7 | 152 | 79 (0, 200) | 159 | 82 (0, 200) |
|  |  | 8 | 150 | 86 (0, 157) | 158 | 78 (0, 189) |
|  |  | 9 | 148 | 87 (0, 179) | 158 | 77 (10, 201) |
|  |  | 10 | 151 | 83 (0, 186) | 157 | 80 (1, 193) |
|  |  | 11 | 145 | 85 (0, 169) | 156 | 76 (0, 210) |
|  |  | 12 | 147 | 86 (0, 180) | 157 | 75 (0, 177) |
|  |  | 13 | 148 | 82 (0, 186) | 157 | 79 (0, 188) |
|  |  | 14 | 145 | 82 (0, 175) | 154 | 73 (0, 169) |
|  |  | 15 | 143 | 84 (0, 217) | 156 | 77 (0, 178) |
|  |  | 16 | 142 | 80 (0, 187) | 155 | 74 (4, 167) |
|  |  | 17 | 147 | 80 (0, 184) | 155 | 79 (7, 186) |
|  |  | 18 | 148 | 88 (0, 248) | 156 | 75 (1, 210) |
|  |  | 19 | 147 | 81 (0, 168) | 156 | 76 (4, 183) |
|  |  | 20 | 143 | 79 (0, 146) | 152 | 78 (6, 201) |
|  |  | 21 | 143 | 77 (0, 174) | 151 | 80 (0, 197) |
|  |  | 22 | 146 | 77 (0, 178) | 148 | 76 (5, 195) |
|  |  | 23 | 144 | 77 (0, 166) | 146 | 79 (0, 179) |
|  |  | 24 | 140 | 78 (0, 177) | 140 | 80 (14, 212) |
|  |  | 25 | 125 | 70 (0, 156) | 136 | 73 (0, 197) |

Table 22. Mean daily number of steps and of minutes spent doing activity (e.g. walking) by week and treatment arm (N= number of patients)

|  | | | **Arm A** | | **Arm B** | |
| --- | --- | --- | --- | --- | --- | --- |
| **Endpoint** | **Measurement** | **Week** | **N** | **Median (range)** | **N** | **Median (range)** |
| Walking activity | Mean daily number of steps | 1 | 151 | 7335 (0, 16671) | 155 | 6834 (0, 24886) |
|  |  | 2 | 155 | 9496 (0, 17834) | 158 | 7901 (13, 23137) |
|  |  | 3 | 156 | 9276 (305, 22049) | 159 | 8370 (118, 21519) |
|  |  | 4 | 156 | 8807 (0, 18047) | 161 | 7986 (630, 23369) |
|  |  | 5 | 154 | 9002 (0, 18036) | 159 | 8069 (43, 20481) |
|  |  | 6 | 150 | 8632 (0, 18707) | 158 | 8251 (106, 21564) |
|  |  | 7 | 152 | 8214 (0, 18977) | 159 | 7977 (751, 21007) |
|  |  | 8 | 150 | 8751 (0, 19231) | 158 | 7942 (608, 21254) |
|  |  | 9 | 148 | 9002 (0, 19633) | 158 | 7805 (683, 20222) |
|  |  | 10 | 151 | 8046 (1518, 19331) | 157 | 8096 (547, 17295) |
|  |  | 11 | 145 | 8471 (1566, 19604) | 156 | 7872 (275, 20656) |
|  |  | 12 | 147 | 8706 (1530, 18511) | 157 | 7739 (463, 19334) |
|  |  | 13 | 148 | 8392 (0, 17754) | 157 | 7692 (163, 21189) |
|  |  | 14 | 145 | 8289 (1431, 16832) | 154 | 7924 (664, 17100) |
|  |  | 15 | 143 | 8551 (0, 20007) | 156 | 7660 (514, 19826) |
|  |  | 16 | 142 | 8036 (352, 20566) | 155 | 7332 (663, 18538) |
|  |  | 17 | 147 | 8143 (208, 18690) | 155 | 7676 (593, 18947) |
|  |  | 18 | 148 | 8699 (0, 25032) | 156 | 7451 (1130, 21603) |
|  |  | 19 | 147 | 8526 (75, 17607) | 156 | 7543 (428, 19220) |
|  |  | 20 | 143 | 8002 (296, 16951) | 152 | 7780 (659, 21306) |
|  |  | 21 | 143 | 8238 (197, 17570) | 151 | 7750 (768, 19068) |
|  |  | 22 | 146 | 7680 (546, 18020) | 148 | 7281 (592, 18494) |
|  |  | 23 | 144 | 8104 (7, 18898) | 146 | 7506 (24, 19382) |
|  |  | 24 | 140 | 7720 (0, 18224) | 140 | 7498 (1087, 19864) |
|  |  | 25 | 125 | 6943 (5, 17129) | 136 | 6650 (290, 20490) |
|  | Mean number of minutes spent doing activity | 1 | 151 | 70 (0, 236) | 155 | 74 (0, 216) |
|  |  | 2 | 155 | 92 (0, 253) | 158 | 82 (0, 207) |
|  |  | 3 | 156 | 88 (3, 182) | 159 | 85 (1, 187) |
|  |  | 4 | 156 | 86 (0, 212) | 161 | 80 (8, 207) |
|  |  | 5 | 154 | 88 (0, 170) | 159 | 85 (0, 184) |
|  |  | 6 | 150 | 87 (0, 240) | 158 | 83 (1, 199) |
|  |  | 7 | 152 | 81 (0, 186) | 159 | 79 (10, 191) |
|  |  | 8 | 150 | 87 (0, 186) | 158 | 81 (7, 193) |
|  |  | 9 | 148 | 90 (0, 186) | 158 | 80 (9, 201) |
|  |  | 10 | 151 | 80 (19, 176) | 157 | 84 (7, 181) |
|  |  | 11 | 145 | 84 (17, 313) | 156 | 80 (3, 204) |
|  |  | 12 | 147 | 83 (19, 173) | 157 | 80 (5, 171) |
|  |  | 13 | 148 | 82 (0, 180) | 157 | 81 (1, 188) |
|  |  | 14 | 145 | 81 (15, 149) | 154 | 80 (7, 162) |
|  |  | 15 | 143 | 80 (0, 217) | 156 | 80 (6, 183) |
|  |  | 16 | 142 | 79 (4, 188) | 155 | 76 (8, 173) |
|  |  | 17 | 147 | 78 (2, 291) | 155 | 82 (7, 178) |
|  |  | 18 | 148 | 87 (0, 247) | 156 | 78 (14, 202) |
|  |  | 19 | 147 | 82 (1, 302) | 156 | 78 (6, 196) |
|  |  | 20 | 143 | 81 (2, 193) | 152 | 81 (8, 197) |
|  |  | 21 | 143 | 80 (2, 177) | 151 | 82 (9, 182) |
|  |  | 22 | 146 | 76 (5, 172) | 148 | 77 (8, 184) |
|  |  | 23 | 144 | 79 (0, 164) | 146 | 80 (0, 185) |
|  |  | 24 | 140 | 80 (0, 216) | 140 | 79 (14, 194) |
|  |  | 25 | 125 | 68 (0, 151) | 136 | 69 (0, 194) |

Table 23. Patients walking 30 minutes for at least 5 days per week during follow up

|  | **FU 1 (N=309)** | **FU 2 (N=304)** |
| --- | --- | --- |
| **Patient walking 30 minutes for at least 5 days per week** | **n (%)** | **n (%)** |
| Arm A |  |  |
| . No | 93 (60.4%) | 107 (71.3%) |
| . Yes | 61 (39.6%) | 43 (28.7%) |
| Arm B |  |  |
| . No | 93 (60.0%) | 113 (73.4%) |
| . Yes | 62 (40.0%) | 41 (26.6%) |

Table 24. Median physical activity by employment status (n=number of patients)

| **Variable** | **Working full-time (N=44)** | | | **Working part-time (N=96)** | | |
| --- | --- | --- | --- | --- | --- | --- |
|  | **n** | **median** | **(min, max)** | **n** | **median** | **(min, max)** |
| Median daily number of steps | 43 | 9166.0 | (1895.0, 15772) | 96 | 9074.0 | (2531.0, 18540) |
| Mean daily number of steps | 43 | 9194.9 | (2001.6, 15952) | 96 | 9063.3 | (2484.9, 17606) |
| Median number of minutes spent doing activity | 43 | 85.9 | (23.6, 169.0) | 96 | 90.2 | (30.9, 182.8) |
| Mean number of minutes spent doing activity | 43 | 89.0 | (24.9, 163.9) | 96 | 91.2 | (29.0, 161.8) |

**Table 35 (continued)**

| **Variable** | **Unemployed (N=43)** | | | **Retired (N=116)** | | | **Other (N=16)** | | |
| --- | --- | --- | --- | --- | --- | --- | --- | --- | --- |
|  | **n** | **median** | **(min, max)** | **n** | **median** | **(min, max)** | **n** | **median** | **(min, max)** |
| Median daily number of steps | 43 | 8270.5 | (2640.0, 14992) | 116 | 7323.5 | (0.0, 16978) | 16 | 5592.5 | (2279.5, 9639.0) |
| Mean daily number of steps | 43 | 8119.8 | (2741.6, 15009) | 116 | 7352.3 | (215.5, 16066) | 16 | 5814.4 | (3047.1, 8445.5) |
| Median number of minutes spent doing activity | 43 | 83.3 | (32.0, 142.5) | 116 | 72.8 | (0.0, 158.1) | 16 | 59.9 | (21.2, 91.9) |
| Mean number of minutes spent doing activity | 43 | 80.4 | (32.9, 134.2) | 116 | 72.8 | (1.9, 153.3) | 16 | 60.9 | (33.1, 83.9) |


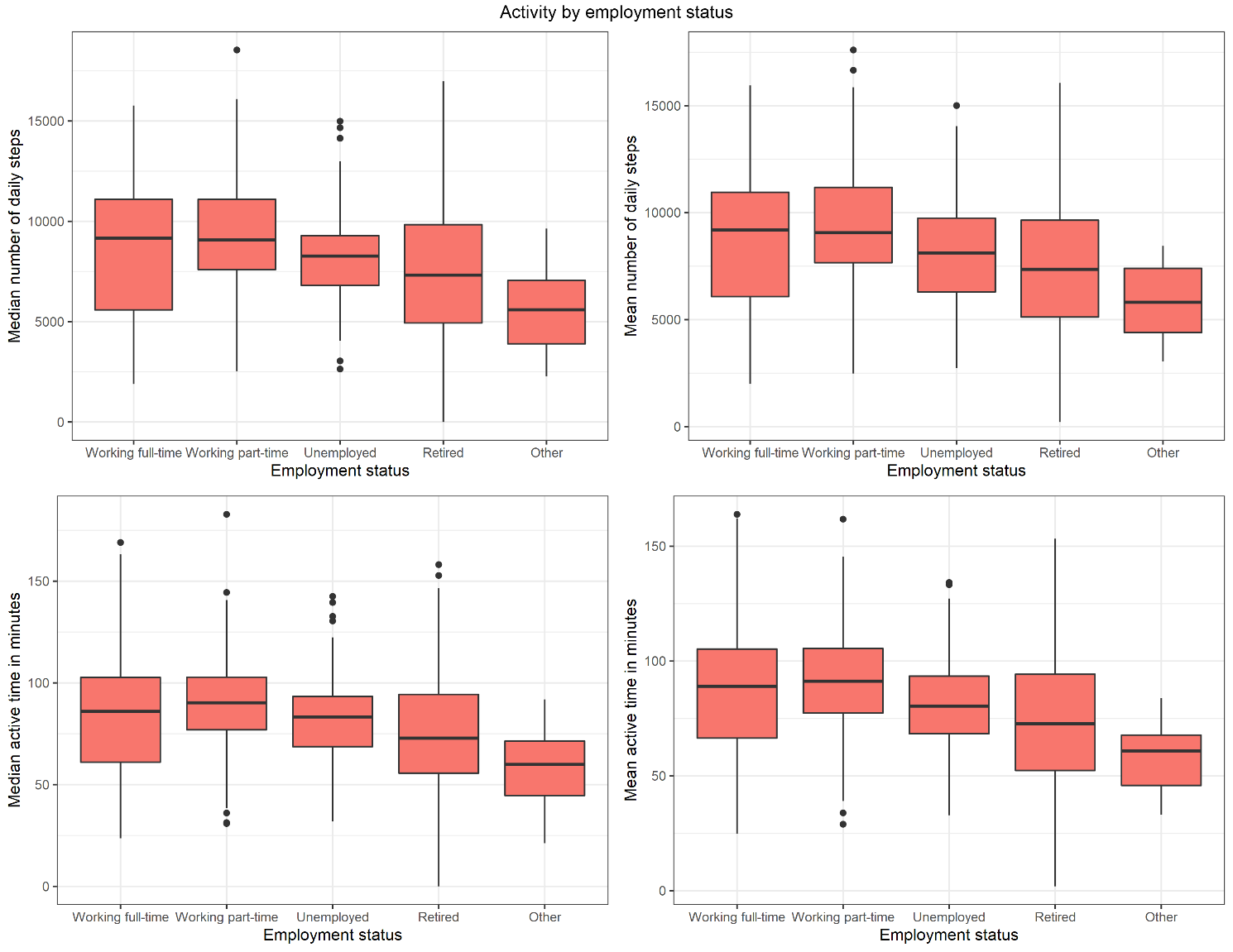


Figure 8. Activity by employment status

Table 25. Physical activity by smoking status (n=number of patients)

| **Variable** | **I am a smoker (N=28)** | | | **I smoke occasionally (no (N=10)** | | | **I am a non-smoker (N=276)** | | |
| --- | --- | --- | --- | --- | --- | --- | --- | --- | --- |
|  | **n** | **median** | **(min, max)** | **n** | **median** | **(min, max)** | **n** | **median** | **(min, max)** |
| Median daily number of steps | 28 | 7197.0 | (1419.5, 15751) | 10 | 7475.8 | (4165.0, 11726) | 275 | 8184.0 | (0.0, 18540) |
| Mean daily number of steps | 28 | 7149.6 | (1924.5, 15952) | 10 | 6819.9 | (4221.6, 11340) | 275 | 8285.8 | (215.5, 17606) |
| Median number of minutes spent doing activity | 28 | 70.8 | (17.8, 163.2) | 10 | 72.2 | (42.6, 121.4) | 275 | 83.4 | (0.0, 182.8) |
| Mean number of minutes spent doing activity | 28 | 71.7 | (22.3, 162.2) | 10 | 69.1 | (48.2, 122.3) | 275 | 83.1 | (1.9, 163.9) |


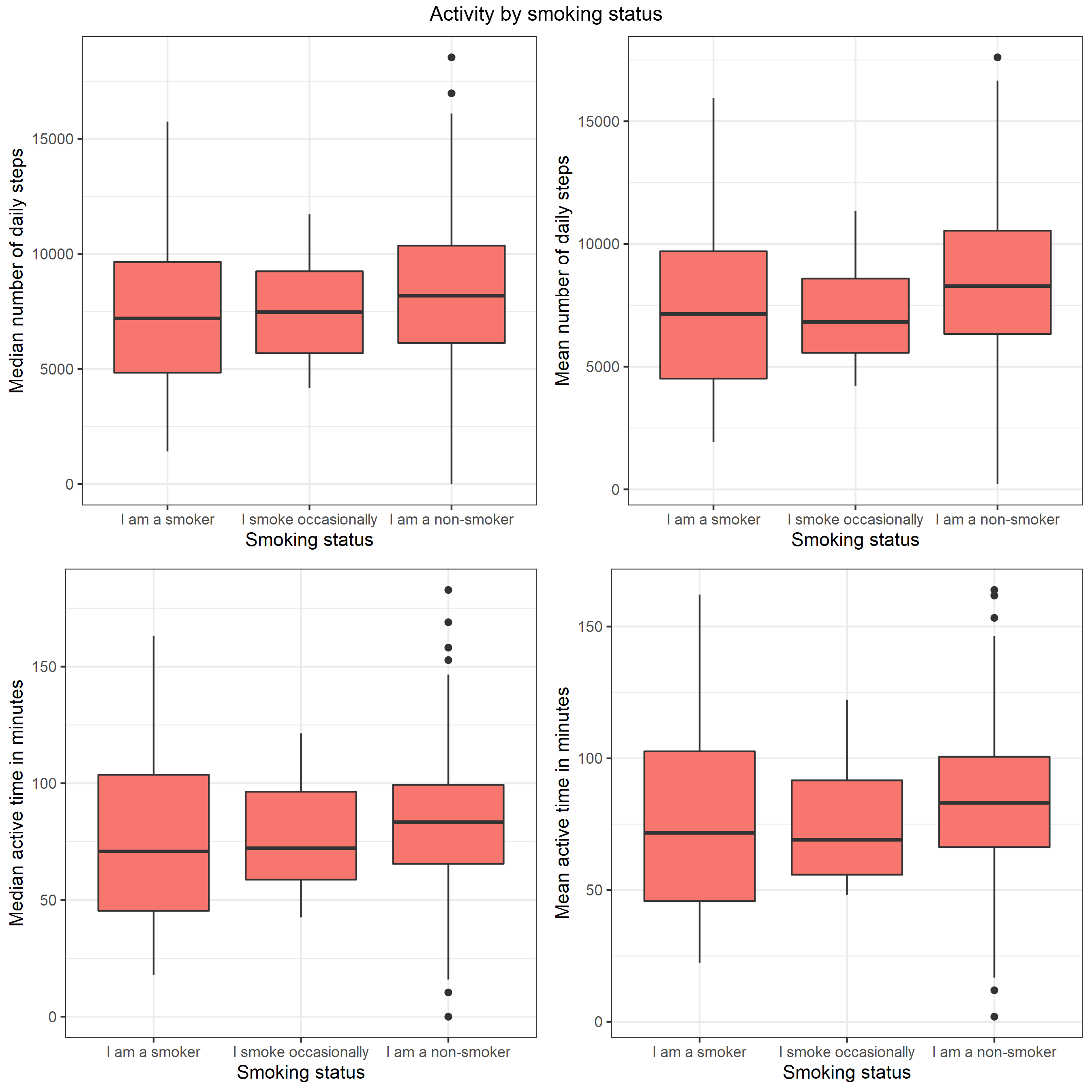


Figure 9. Activity by smoking status

Table 26. Physical activity by marital status (n=number of patients). The following statement was assessed: “I live in a partnership and I live together with my partner”.

| **Variable** | **Is not true (N=79)** | | | **Is true (N=235)** | | |
| --- | --- | --- | --- | --- | --- | --- |
|  | **n** | **median** | **(min, max)** | **n** | **median** | **(min, max)** |
| Median daily number of steps | 79 | 8069.5 | (0.0, 14143) | 234 | 8074.5 | (805.0, 18540) |
| Mean daily number of steps | 79 | 8121.8 | (215.5, 13619) | 234 | 8184.7 | (950.2, 17606) |
| Median number of minutes spent doing activity | 79 | 78.0 | (0.0, 142.5) | 234 | 83.1 | (10.4, 182.8) |
| Mean number of minutes spent doing activity | 79 | 78.1 | (1.9, 140.6) | 234 | 81.9 | (11.9, 163.9) |


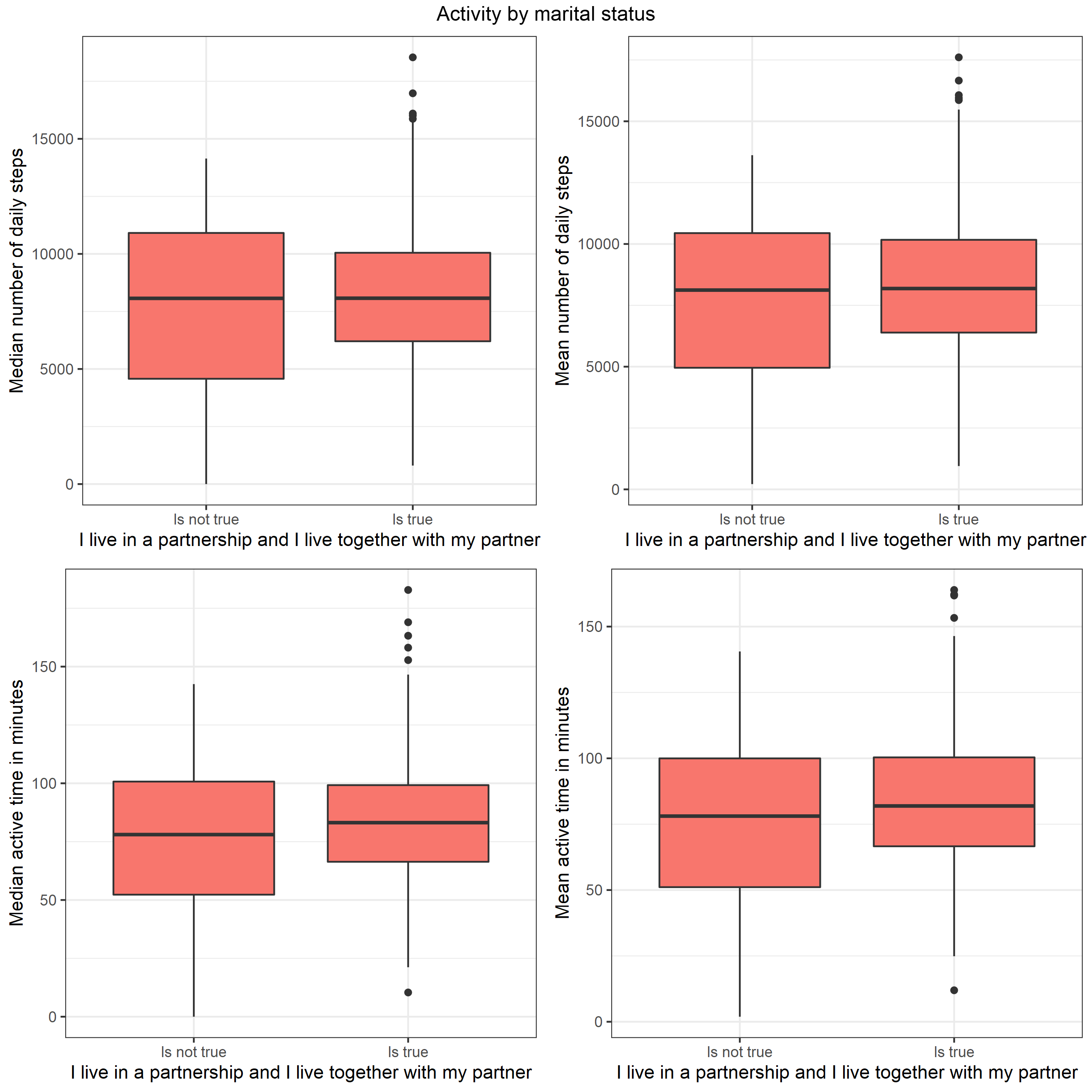


Figure 10. Activity by marital status

Table 27. Daily numer of steps and minutes spent doing activity during the whole intervention phase

| **Variable** | **Arm A (N=158)** | | | **Arm B (N=162)** | | |
| --- | --- | --- | --- | --- | --- | --- |
|  | **n** | **median** | **(min, max)** | **n** | **median** | **(min, max)** |
| Mean daily number of steps | 158 | 8292.0 | (215.5, 16660) | 161 | 8035.8 | (950.2, 17606) |
| Median daily number of steps | 158 | 8541.5 | (0.0, 16099) | 161 | 7742.0 | (805.0, 18540) |
| Mean number of minutes spent doing activity | 158 | 82.1 | (1.9, 162.2) | 161 | 81.3 | (11.9, 163.9) |
| Median number of minutes spent doing activity | 158 | 83.1 | (0.0, 163.2) | 161 | 81.3 | (10.4, 182.8) |

#### General physical activity

During trial intervention phase and during follow-up phase at 12 and 24 months (± 61 days), physical activity was measured by the GPAQ, a patient self-report questionnaire developed by the WHO for physical activity surveillance on a country level. The 16-item questionnaire collects information on physical activity participation in three settings (or domains) as well as sedentary behavior. The domains are: activity at work, travel to and from places, and recreational activities. Calculations were performed according to the GPAQ guide for analysis and the result is shown in Table 28. Additionally, the number of MET-minutes/week as a measure of how much energy has been expended during a week is summarized in Table 29 and Figure 11.

Table 28. Physical activity measured by the GPAQ

|  | **Arm A Intervention (N=158)** | **Arm B Non-Intervention (N=162)** |
| --- | --- | --- |
| **Variable** | **n (%)** | **n (%)** |
| Baseline |  |  |
| . Active | 119 (75.3%) | 131 (80.9%) |
| . Inactive | 24 (15.2%) | 21 (13.0%) |
| . Missing | 15 (9.5%) | 10 (6.2%) |
| Week 13 |  |  |
| . Active | 124 (78.5%) | 127 (78.4%) |
| . Inactive | 9 (5.7%) | 13 (8.0%) |
| . Missing | 25 (15.8%) | 22 (13.6%) |
| Week 25 |  |  |
| . Active | 125 (79.1%) | 118 (72.8%) |
| . Inactive | 7 (4.4%) | 15 (9.3%) |
| . Missing | 26 (16.5%) | 29 (17.9%) |
| Follow-up 1 |  |  |
| . Active | 125 (79.1%) | 113 (69.8%) |
| . Inactive | 6 (3.8%) | 14 (8.6%) |
| . Missing | 27 (17.1%) | 35 (21.6%) |
| Follow-up 2 |  |  |
| . Active | 110 (69.6%) | 99 (61.1%) |
| . Inactive | 6 (3.8%) | 17 (10.5%) |
| . Missing | 42 (26.6%) | 46 (28.4%) |

Table 29. Summary of the number of MET-minutes/week by treatment arm and visit

| **Variable** | **Arm A Intervention (N=158)** | | | **Arm B Non-Intervention (N=162)** | | |
| --- | --- | --- | --- | --- | --- | --- |
|  | **n** | **median** | **(min, max)** | **n** | **median** | **(min, max)** |
| MET-minutes/week at baseline | 143 | 2320.0 | (0.0, 46080) | 152 | 2360.0 | (0.0, 43200) |
| MET-minutes/week at week 13 | 133 | 2920.0 | (0.0, 31080) | 140 | 2400.0 | (0.0, 26880) |
| MET-minutes/week at week 25 | 132 | 2640.0 | (0.0, 41280) | 133 | 3420.0 | (0.0, 84000) |
| MET-minutes/week at follow-up 1 | 131 | 3440.0 | (0.0, 34560) | 127 | 3120.0 | (0.0, 24960) |
| MET-minutes/week at follow-up 2 | 116 | 3720.0 | (0.0, 43200) | 116 | 2730.0 | (0.0, 30480) |


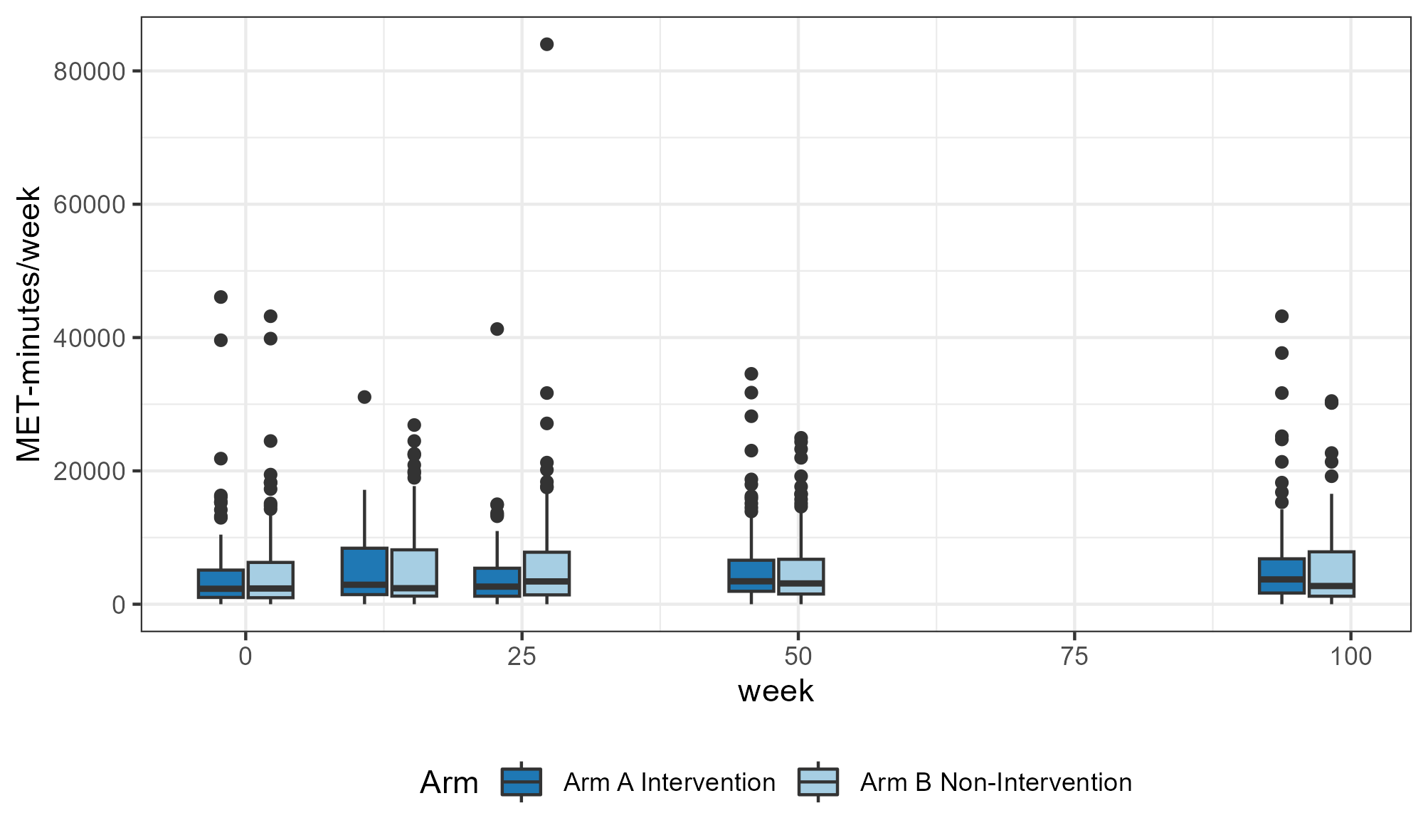


Figure 11. Boxplot summarizing of the number of MET-minutes/week by treatment arm and visit

#### Weight and BMI

Absolute and relative changes from baseline in weight and BMI were calculated during the trial intervention phase. The results can be found in Table 30.

Table 30. Absolute and relative changes from baseline in weight and BMI

| **Variable** | **Arm A Intervention (N=158)** | | | **Arm B Non-Intervention (N=162)** | | |
| --- | --- | --- | --- | --- | --- | --- |
|  | **n** | **median** | **(min, max)** | **n** | **median** | **(min, max)** |
| Absolute change in weight from baseline to week 13 | 136 | -0.1 | (-7.0, 7.0) | 142 | 0.0 | (-12.0, 7.0) |
| Relative change in weight from baseline to week 13 (%) | 136 | -0.1 | (-10.3, 8.9) | 142 | 0.0 | (-14.0, 9.4) |
| Absolute change in weight from baseline to week 25 | 140 | 0.0 | (-10.6, 11.0) | 140 | 0.0 | (-9.0, 14.0) |
| Relative change in weight from baseline to week 25 (%) | 140 | 0.0 | (-12.5, 17.7) | 140 | 0.0 | (-10.2, 18.2) |
| Absolute change in BMI from baseline to week 13 | 135 | 0.0 | (-2.6, 2.6) | 142 | 0.0 | (-4.0, 3.1) |
| Relative change in BMI from baseline to week 13 (%) | 135 | 0.0 | (-10.3, 8.9) | 142 | 0.0 | (-14.0, 9.4) |
| Absolute change in BMI from baseline to week 25 | 140 | 0.0 | (-4.1, 3.7) | 140 | 0.0 | (-3.2, 6.2) |
| Relative change in BMI from baseline to week 25 (%) | 140 | 0.0 | (-12.5, 17.7) | 140 | 0.0 | (-10.2, 18.2) |
